# Structure‐Function Relationships of Biomass‐Derived Materials for Zinc Metal Protection

**DOI:** 10.1002/advs.202507768

**Published:** 2025-07-15

**Authors:** Qiwei Gao, Zhuo Chen, Ya He, Zhangxiang Hao, Junrun Feng

**Affiliations:** ^1^ School of Science School of Chip Industry Hubei University of Technology Wuhan Hubei 430068 China

**Keywords:** aqueous zinc metal batteries, biomass‐derived materials, functional groups, interface engineering, synergistic effects

## Abstract

Aqueous zinc‐ion batteries have attracted significant attention for large‐scale energy storage applications, yet their practical implementation is fundamentally limited by zinc anode stability. This review first establishes a comprehensive understanding of zinc degradation mechanisms, revealing how ion transport, solvation dynamics, and electrical double layer structure collectively determine interfacial stability. The fundamental properties and protective mechanisms of biomass‐derived materials are systematically analyzed, focusing on four key functional groups: carboxyl, amino, hydroxyl, and sulfonic acid. Through examining their roles in regulating critical interfacial processes, quantitative structure‐function relationships are established that reveal optimal interface protection requires balanced binding energies rather than maximizing individual interactions. More significantly, it is demonstrated that spatial distribution and synergistic effects among multiple functional groups enable superior interface regulation beyond simple additive benefits. These molecular‐level insights transform empirical additive selection into rational design principles. Critical challenges in mechanistic understanding, scalability, and standardization are identified, proposing strategic directions for advancing zinc‐based energy storage technologies.

## Graphical abstract

This review analyses the mechanism of zinc anode degradation, focuses on the protective roles of the four major functional groups of carboxyl, amino, hydroxyl, and sulfonic acid groups in biomass materials, reveals the structure‐function relationship and synergistic effects of functional groups, and looks forward to the challenges and directions of zinc‐based energy storage technology.

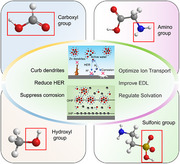

## 1 Introduction

The transition toward renewable energy has sparked an urgent quest for safe, sustainable energy storage solutions.^[^ ^1^, ^2^, ^3^, ^4^ ^]^ While lithium‐ion batteries dominate portable electronics, their high cost ($135 kWh^−1^), safety concerns, and environmental impact limit their viability for grid‐scale storage.^[^ ^5^, ^6^, ^7^, ^8^ ^]^ This challenge has driven intense interest in aqueous zinc‐ion batteries (AZIBs), which offer compelling advantages through their high volumetric capacity (5855 mAh cm^−3^), intrinsically safe aqueous electrolytes, and abundant raw materials.^[^ ^9^, ^10^, ^11^ ^]^ More significantly, zinc's high recyclability and low cost ($25 kWh^−1^) position AZIBs as an environmentally and economically attractive solution for large‐scale energy storage.^[^ ^12^, ^13^ ^]^

However, the practical implementation of AZIBs is fundamentally limited by the intrinsic instability of zinc metal in aqueous environments.^[^ ^14^, ^15^ ^]^ When the zinc anode contacts with aqueous electrolyte, the negative electrode potential (−0.76V vs. SHE) triggers inevitable water decomposition, generating hydrogen gas and creating local pH fluctuations at the electrode‐electrolyte interface.^[^ ^16^, ^17^, ^18^ ^]^ This interfacial instability is further complicated by the unique Zn^2+^ solvation behavior [Zn(H_2_O)_6_]^2+^ and creates significant energy barriers during the plating process.^[^ ^19^, ^20^, ^21^, ^22^ ^]^ The inhomogeneous electric field distribution and uneven ion flux near the electrode surface lead to preferential zinc deposition, eventually growing dendrites that penetrate the separator.^[^ ^23^, ^24^, ^25^ ^]^ Meanwhile, the continuous reaction between zinc and water molecules forms insulating passive layers (primarily Zn_4_SO_4_(OH)_6_·xH_2_O (ZHS)), which not only increase the interfacial resistance but also crack under repeated volume changes during cycling.^[^ ^26^, ^27^ ^]^ The cracking exposes fresh zinc surface, accelerating the degradation process. These interlinked degradation mechanisms create a complex failure cascade that severely compromises the battery performance and lifetime.^[^ ^28^, ^29^, ^30^ ^]^

Conventional protection strategies, including inorganic coatings and organic modifiers, have struggled to simultaneously address these interconnected interfacial challenges.^[^ ^31^, ^32^, ^33^ ^]^ While inorganic protective layers (e.g., ZnO, TiO_2_) can temporarily suppress hydrogen evolution reaction (HER), their poor flexibility leads to cracking under repeated volume changes. Similarly, synthetic organic modifiers show promising initial performance but rapidly degrade in the strongly acidic/alkaline environment (pH 4–11) near the electrode surface. Moreover, the high cost of these materials (e.g. Battery grade ZnO, priced at $42–85 kg^−1^; Battery grade TiO_2_ at $40–80 kg^−1^; Tetraethylammonium bromide (TEAB), $208 kg^−1^) and their environmental impact limit practical implementation.^[^ ^34^, ^35^, ^36^, ^37^, ^38^, ^39^ ^]^

**Figure 1:**
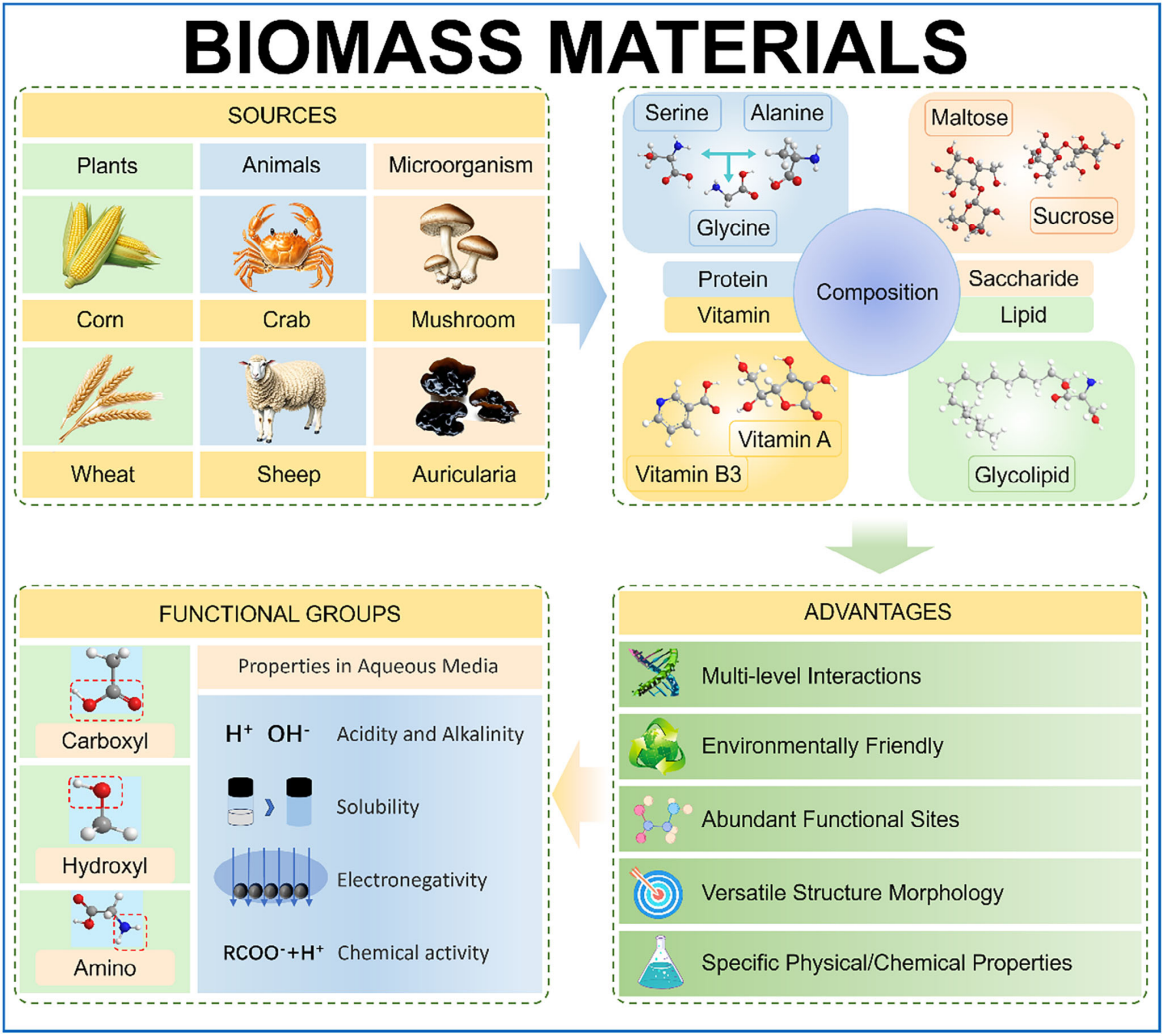
Schematic illustration of biomass materials, presenting their sources, compositional components, prominent advantages and key functional groups with properties in aqueous media.

Nature offers an inspiring solution through biomass materials, which have evolved sophisticated multi‐level protection mechanisms (**Figure** **1**).^[^ ^40^ ^]^ These materials present a unique combination of hierarchical structures and diverse functional groups including carboxyl, amino, hydroxyl, and sulfonic acid groups that could revolutionize zinc protection strategies.^[^ ^41^, ^42^ ^]^ Their hierarchical architecture spans from molecular to microscale, enabling comprehensive interface regulation while maintaining environmental sustainability. Moreover, these materials derived from agricultural wastes offer dramatic cost advantages with significantly lower carbon footprint.^[^ ^43^, ^44^ ^]^ The Figure S1 and Table S3 (Supporting Information) clearly show that biomass materials are far less expensive than other commonly used materials.

Scientists and researchers have made significant advances in employing biomass materials for zinc anode protection, and these biomass materials show strong performance in suppressing hydrogen evolution, guiding zinc deposition, and stabilizing the electrode–electrolyte interface.^[^ ^40^, ^42^, ^43^, ^45^ ^]^ For instance, Chen et al. utilized cellulose nanocrystals (CNCs) with high‐aspect‐ratio nanofibers to achieve stable cycling for 982 h at 50 mA cm^−2^, leveraging a synergistic mechanism of chemical coordination and physical guidance.^[^ ^46^ ^]^ Inspired by bamboo's porous architecture, Kuang et al. incorporated bamboo thin‐walled cell (BPC) additives, enabling 3000 h of stable cycling at 2 mA cm^−2^. Both CNCs and BPCs exploit the hierarchical features of biomass, from functional groups to pore structures, to regulate Zn^2+^ solvation, deposition, and interfacial reactions. In addition to their electrochemical benefits, CNCs are biodegradable, and BPCs are derived from agricultural waste, aligning with sustainable energy goals. This bio‐inspired approach offers a promising direction for both fundamental research and practical application in zinc‐based energy storage systems.^[^ ^47^ ^]^

The unique structure of the biomass material also has an important effect on the Zn anode performance. In cellulose‐based materials, cellulose is rich in hydroxyl groups and other functional groups, and cellulose‐based gel electrolytes made by modification can complex with Zn^2+^ in aqueous zinc ion batteries, regulate the solvation structure of Zn^2+^, and promote the uniform deposition of Zn^2+^ in order to inhibit the dendritic crystal growth, like the gel electrolyte prepared by composite of carboxymethylcellulose sodium (CMC) and polyvinyl alcohol (PVA), which can reduce interfacial impedance and make Zn electrode cycle at 1 mA cm^−2^ current density.^[^ ^48^ ^]^ The gel electrolyte prepared by sodium CMC and PVA composite can reduce the interfacial impedance, so that the Zn electrode maintains a stable Coulombic efficiency after 1000 h of cycling at a current density of 1 mA cm^−2^, and improves the cycling performance and stability of the battery; and because of its 3D reticular structure and a large number of active sites, the lignin‐based carbon material prepared by pyrolysis and oxidation can provide abundant nucleation sites as a protective coating for the Zn electrode.^[^ ^49^ ^]^ When the lignin‐based carbon material prepared by pyrolysis and oxidation is used as the protective coating for Zn electrode, its structure can provide abundant nucleation sites, guide the uniform deposition of Zn^2+^, avoid the formation of dendrites, and the high conductivity helps to reduce the charge transfer impedance. In practical application, the Zn electrode coated with this material can achieve uniform Zn deposition at a high current density of 5 mA cm^−2^, and the capacity retention rate of the battery is more than 90% after 100 times of cycling, which improves the performance of the battery significantly.

While significant progress has demonstrated the promise of biomass materials in zinc protection, establishing fundamental structure‐function relationships remains essential for rational design and optimization. Herein, we systematically analyze the structure‐function relationships of biomass‐derived materials for zinc protection. We first examine the fundamental mechanisms of zinc degradation in aqueous environments and evaluate the role of different functional groups in interface regulation. Special attention is given to the interaction between functional groups and zinc ions, including binding energy calculations and experimental validations. We then discuss how the spatial distribution and density of functional groups affect their protective efficiency, supported by both theoretical analysis and experimental evidence. The synergistic effects between different types of functional groups are thoroughly investigated, providing insights into their cooperative protection mechanisms. Based on these analyses, we propose practical guidelines for optimizing biomass‐derived materials in zinc protection, considering factors such as functional group selection, structural design, and processing methods. Finally, we outline the remaining challenges and future opportunities in this field, particularly focusing on understanding the long‐term stability mechanisms and scaling up production while maintaining performance. This comprehensive analysis not only advances our understanding of biomass materials in zinc protection but also provides valuable insights for developing sustainable energy storage solutions.

## 2 Fundamental Understanding of Zinc Metal Degradation Mechanisms

**Figure 2:**
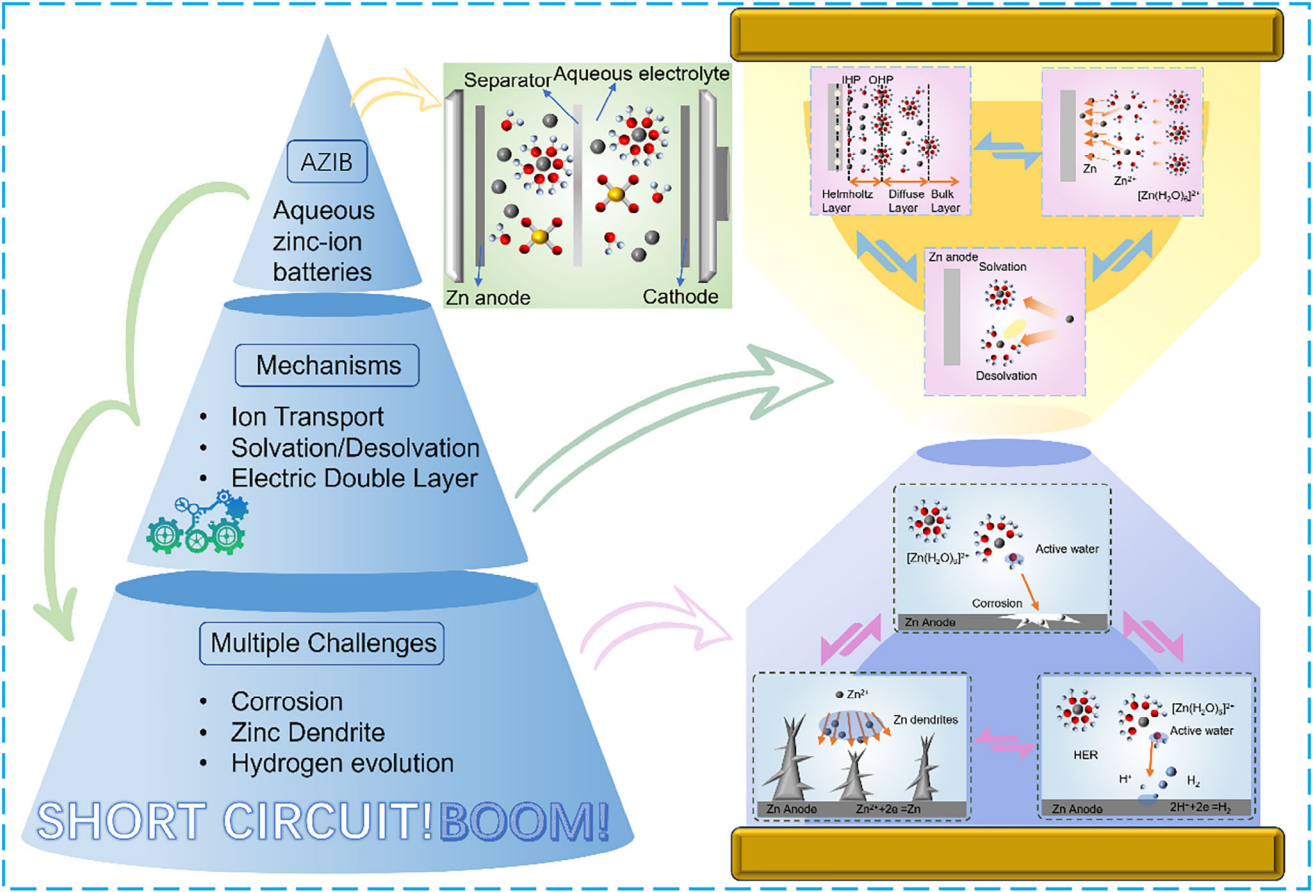
Schematic diagram of the structure and mechanism of AIZBs and the challenges faced by zinc anodes.

Understanding zinc metal degradation in aqueous environments requires systematic analysis from fundamental interfacial behaviors to their coupled failure consequences. This section first examines the basic interfacial structures and transport properties, including ion transport, solvation chemistry, and electrical double layer (EDL) characteristics. These fundamental aspects then manifest as specific degradation phenomena‐hydrogen evolution, corrosion, and dendrite formation. Finally, we analyze how these degradation processes interact and amplify each other, forming complex feedback networks that ultimately determine battery failure modes (**Figure** **2**).

### 2.1 Fundamental Interfacial Behaviors and Properties

Understanding interfacial processes at the Zn metal/electrolyte interface is fundamental to AZIBs. These processes can be systematically analyzed through three interconnected aspects: the physical transport of zinc ions, their solvation structure transitions, and the formed EDL structure at the interface. The transport behavior determines how zinc ions move and deposit, the solvation chemistry controls their reactivity and energy barriers, while the EDL structure governs the local environment for interfacial reactions.^[^ ^50^, ^51^, ^52^ ^]^

Throughout the charging and discharging process, Zn^2+^ performs several sequential steps in the electrolyte. Initially, ion transport occurs, with hydrated cations diffusing through the bulk electrolyte, followed by electric field‐driven migration toward the anode with a series of conversion reactions. Ion transport at the zinc metal/electrolyte interface can be quantitatively described by the Nernst‐Planck equation, which captures three key transport mechanisms (Equation (1)):^[^ ^53^ ^]^

(1) $$J=-\frac{qCD}{KT}\frac{dV}{dx}-D\left(\frac{dC}{dx}\right)+Cv_{x}$$

where zinc ion movement is governed by electromigration $-\frac{qCD}{KT}\frac{dV}{dx}$, diffusion $D(\frac{dC}{dx})$, and convection C*v_x_*. The electromigration term describes charged zinc ion movement under electric fields, particularly significant during high rate charging where strong field gradients develop near the electrode surface.^[^ ^54^ ^]^ The diffusion term, driven by concentration gradients, becomes rate‐limiting when ions are consumed faster than they can be replenished, potentially leading to local depletion regions and dendrite formation. Convection effects, while often secondary in conventional batteries, become significant in systems with engineered electrolyte flow or during rapid charging where local heating induces natural convection.^[^ ^55^, ^56^ ^]^

Experimental investigations have revealed complex transport behaviors beyond classical predictions. In 2 m ZnSO_4_ electrolytes, the measured Zn^2+^ diffusion coefficient (6.2×10^−6^ cm^2^ s^−1^) notably exceeds theoretical predictions from Debye‐Hückel theory (4.8×10^−6^ cm^2^ s^−1^).^[^ ^57^ ^]^ This enhancement stems from the restructuring of water molecules around Zn^2+^ in concentrated solutions, demonstrating how solvation effects fundamentally alter transport dynamics. The local electric field distribution, particularly affected by surface irregularities and electrode morphology, creates preferential deposition sites that can initiate non‐uniform growth.^[^ ^58^ ^]^

**Figure 3:**
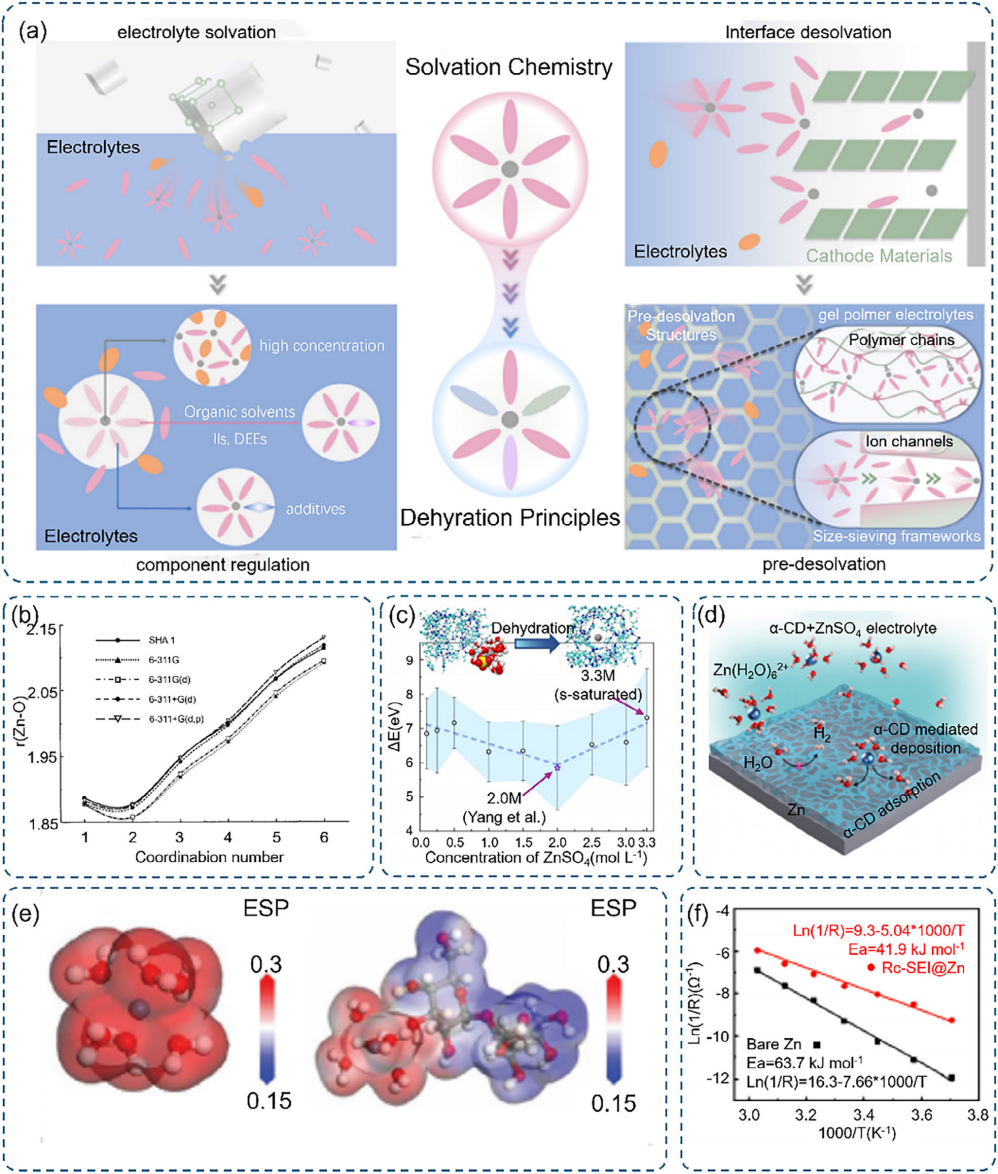
Presents several aspects related to zinc ions in aqueous electrolytes. a) The solvation regulation of zinc ions in aqueous electrolytes, spanning from solvation chemistry to dehydration principles. Reproduced with permission.^[^ ^61^ ^]^ Copyright 2024, Royal Society of Chemistry. b) Depicted is the relationship where the zinc‐oxygen bond length r(Zn─O) [Å] is plotted as a function of the coordination number n. Reproduced with permission.^[^ ^60^ ^]^ Copyright 1997, American Chemical Society. c) The dehydration energy *Δ*E from the bulk to the MOF is presented for concentration gradients ranging from 0.1 m to the saturated 3.3 m. Reproduced with permission.^[^ ^64^ ^]^ Copyright 2023, Wiley‐VCH. d) A schematic illustration demonstrates the Zn plating behaviors in an aqueous ZnSO_4_ electrolyte containing α‐CD. Reproduced with permission.^[^ ^65^ ^]^ Copyright 2022, American Chemical Society. e) Electrostatic potential mapping is carried out, comparing the original Zn^2+^‐6H_2_O with Zn^2+^‐5H_2_O‐trahalose. Reproduced with permission.^[^ ^66^ ^]^ Copyright 2024, Wiley‐VCH. f) Arrhenius curves are shown along with a comparison of activation energies for the desolvation of hydrated zinc ions. Reproduced with permission.^[^ ^54^ ^]^ Copyright 2022, Elsevier.

While the Nernst‐Planck equation describes the macroscopic transport behavior of zinc ions, the microscopic nature of these transport processes is fundamentally determined by their solvation structure (**Figure** **3**a).^[^ ^59^ ^]^ In aqueous electrolytes, Zn^2+^ is transported or migrated mainly as [Zn(H_2_O)_6_]^2+^ with an average Zn─O bond length of 2.0–2.1 Å and a primary hydration shell radius of 2.13 Å (Figure 3b).^[^ ^60^, ^61^, ^62^ ^]^ The substantial formation energy of this hexahydrate (−491 kcal mol^−1^, with Shchez et al. reporting a similar hydration energy of −518 kcal mol^−1^) significantly affects the activation potential for charge transfer.^[^ ^63^ ^]^

The solvation behavior exhibits strong concentration dependence, particularly evident in ZnSO_4_ electrolytes. Systematic investigations across concentrations from dilute (0.1 m) to saturated (3.3 m) solutions reveal optimal properties at 2.0 m, where the system achieves the lowest measured free energy without dehydration (5.84 eV) (Figure 3c).^[^ ^64^ ^]^ This concentration optimum coincides with maximum conductivity and uniform distribution of water‐mediated ion pairs, establishing favorable conditions for Zn^2+^ dehydration. The mechanistic basis for this optimal concentration lies in the balanced competition between ion‐water and ion‐ion interactions, where sufficient ionic strength exists to disrupt bulk water structure without excessive ion clustering (Figure 3d,e).^[^ ^67^, ^68^ ^]^ During the deposition process, [Zn(H_2_O)_6_]^2+^ undergoes sequential transformations at the electrode interface. Initial diffusion brings the solvated complex to the electrode surface, followed by the critical step of partial or complete desolvation to form Zn^2+^, subsequent reduction, and final deposition as metallic Zn.^[^ ^53^, ^59^, ^61^ ^]^ The desolvation process, requiring an activation energy of 63.7 kJ mol^−1^ as determined from Arrhenius analysis, represents a significant kinetic barrier that influences the overall deposition rate and morphology (Figure 3f). Strategic modification of the solvation environment offers multiple pathways for controlling zinc deposition behavior, which will be detailed discussed in Section 3.^[^ ^54^ ^]^

The EDL at the zinc metal/electrolyte interface represents a critical region where solvated zinc ions undergo final transformations before deposition. The formation of this EDL structure is driven by a fundamental thermodynamic process. When metallic zinc contacts the electrolyte, the strong hydration force of Zn^2+^ exceeds the electrostatic attraction from the metal's electron cloud, causing surface Zn^2+^ to transfer into solution.^[^ ^69^, ^70^ ^]^ This spontaneous process, driven by a chemical potential difference of ≈0.76 V (vs. SHE), results in excess electron accumulation at the anode surface, establishing the characteristic negative surface charge.^[^ ^71^, ^72^, ^73^ ^]^

**Figure 4:**
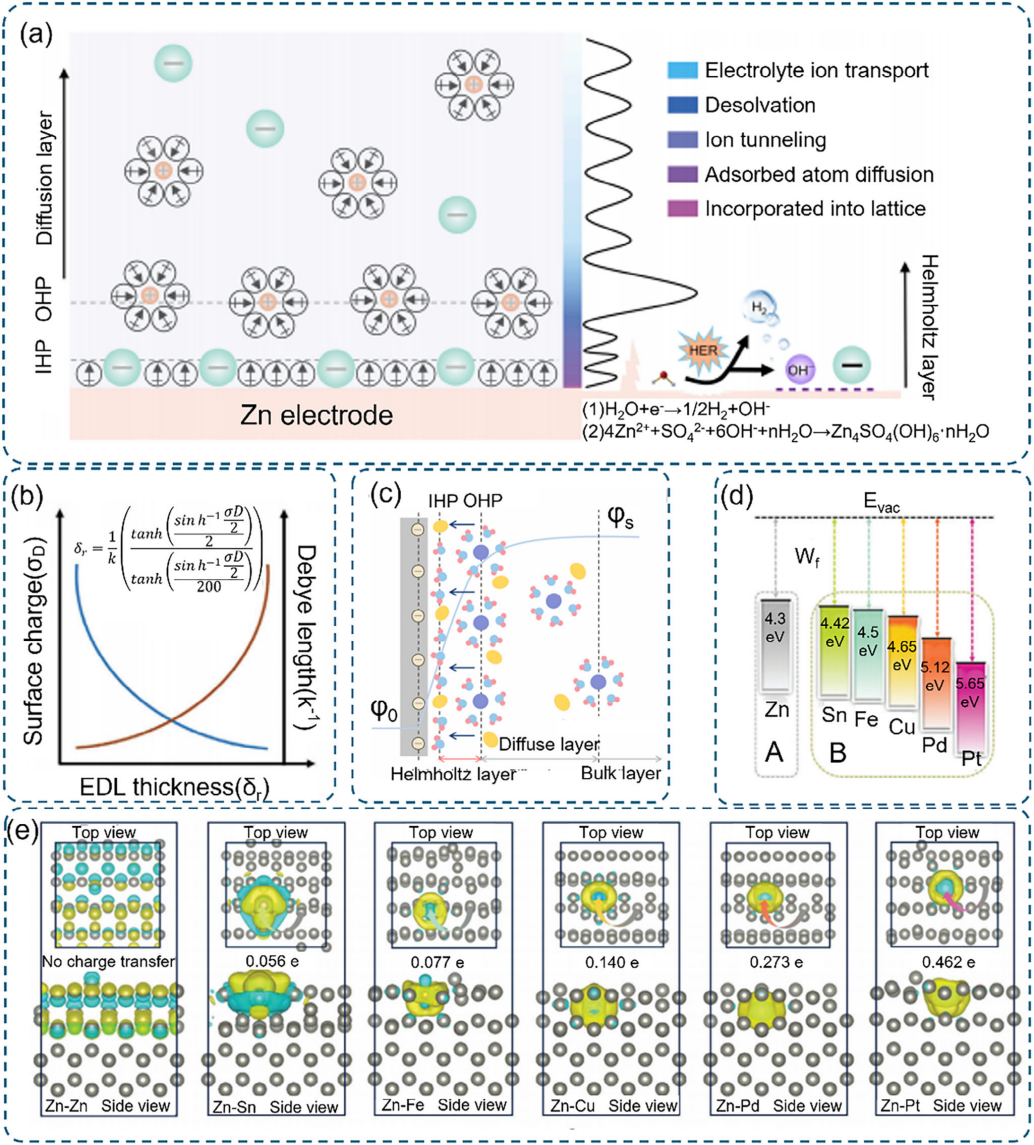
a) Schematic diagrams depicting the EDL structure at the Zn electrode in a ZnSO_4_ aqueous electrolyte system. Reproduced with permission.^[^ ^69^ ^]^ Copyright 2023, Elsevier. b) Variation of EDL thickness as a function of surface charge density and Debye–Hückel length. c) Schematic illustration of the metal/electrolyte interphase. Reproduced with permission.^[^ ^42^ ^]^ Copyright 2024, Elsevier. d) W_f_ values for different metals. e) Calculated charge density differences for Zn, Sn, Fe, Cu, Pd, and Pt atoms adsorbed on the Zn surface, where yellow and blue surfaces denote charge accumulation and depletion, respectively. b), d), and e) Reproduced with permission.^[^ ^81^ ^]^ Copyright 2024, Wiley‐VCH.

The resulting EDL exhibits a complex multilayer structure following the Gouy‐Chapman‐Stern model (**Figure** **4**a).^[^ ^74^ ^]^ The compact layer (CL) closest to the electrode surface subdivides into the inner Helmholtz plane (IHP), containing specifically adsorbed species, and the outer Helmholtz plane (OHP), populated by solvated ions. Beyond these lies the diffusion layer (DL) extending into the bulk electrolyte.^[^ ^75^, ^76^ ^]^ It is noteworthy that under AZIB charging/discharging, EDL can withstand potential changes of several volts within such a thin layer (3–5 Å). This stratified structure creates an exponential potential distribution, with remarkably steep gradients (≈10^8^ V m^−1^) within the first few angstroms, fundamentally affecting interfacial charge transfer processes. Such a large localized electric field leads to highly heterogeneous and dynamic changes in the components and structure of the EDL.^[^ ^71^, ^77^ ^]^

The EDL thickness (δ), described by the Equation (2):

(2) $$\delta_{r}=\frac{1}{k}\left(\frac{\tanh\left(\frac{\sin h^{-1}\frac{\sigma D}{2}}{2}\right)}{\tanh\left(\frac{\sin h^{-1}\frac{\sigma D}{2}}{200}\right)}\right)$$

where δ is the EDL thickness, K⁻¹ is the Debye–Hückel length, and σ is the surface charge density (Figure 4b).^[^ ^78^, ^79^ ^]^ During electrodeposition, the electrode surface potential evolves from ψ_0_ to ψ_s_ as cations adsorb (Figure 4c), with the EDL thickness directly influencing inter‐deposit repulsion forces.^[^ ^80^ ^]^ This understanding has led to strategic EDL engineering approaches, such as incorporating high‐valence cations to compress the EDL structure and electron‐rich functional groups to enhance electrode‐Zn^2+^ interactions (Figure 4d,e).^[^ ^81^ ^]^

The EDL structure significantly influences HER kinetics, a persistent challenge in zinc battery systems. The presence of water dipoles in the IHP, combined with the low Zn/Zn^2+^ redox potential, facilitates HER.^[^ ^82^, ^83^ ^]^ The resulting OH^−^ generation leads to insulating ZHS by‐product formation through reaction with SO_4_^2−^, compromising coulombic efficiency (CE) and battery stability.^[^ ^84^, ^85^, ^86^ ^]^ Moreover, the high desolvation energy barrier of [Zn(H_2_O)_6_]^2+^ at the OHP creates local polarization, increasing overpotential and HER risk.^[^ ^87^ ^]^ Under high‐rate conditions, these desolvation penalties severely impact charge transfer kinetics between the OHP and IHP, promoting non‐uniform ion flux and dendrite formation.^[^ ^88^ ^]^

The interfacial processes at the Zn metal/electrolyte interface emerge from the coupling effects between ion transport, solvation dynamics, and EDL characteristics.^[^ ^89^ ^]^ These three aspects are inherently interconnected: the solvation structure determines the energy barriers for charge transfer, the EDL architecture controls local ion distribution, while transport phenomena govern the overall kinetics of interfacial reactions.^[^ ^51^, ^90^, ^91^ ^]^

The coupling of these processes creates several critical challenges that lead to interface degradation.^[^ ^92^ ^]^ Surface heterogeneities create non‐uniform electric fields and preferential transport pathways, while the high desolvation energy barrier at the OHP‐IHP interface promotes side reactions.^[^ ^93^, ^94^ ^]^ These fundamental processes significantly influence the interface stability and evolution during battery operation, which will be discussed in detail in the following section.

### 2.2 Interface Degradation Phenomena

The complex interfacial processes discussed above, which are ion transport, solvation dynamics, and EDL structure, inevitably lead to several degradation phenomena during battery operation.^[^ ^95^, ^96^ ^]^ Among these, HER, corrosion, and dendrite formation represent the most critical challenges that significantly impact battery performance and stability.^[^ ^97^, ^98^ ^]^

**Figure 5:**
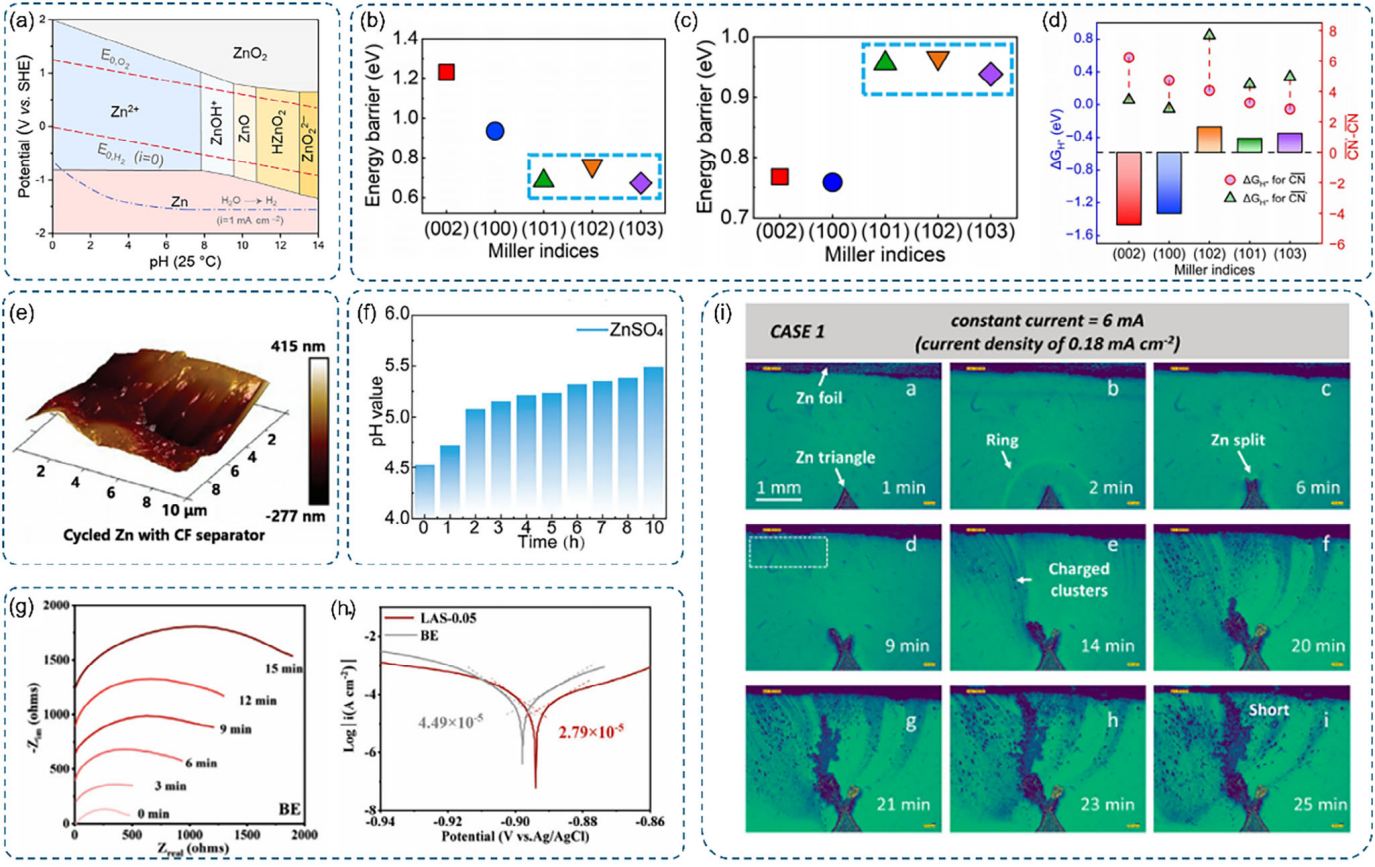
a) Pourbaix diagram illustrating the electrochemical stability of Zn in aqueous solutions. Reproduced with permission.^[^ ^42^ ^]^ Copyright 2024, Elsevier. b) Comparative barriers for the Volmer reaction across different conditions. c) Comparative barriers for the Tafel reaction under varying scenarios. d) Hydrogen adsorption energy of H species adsorbed on surface Zn atoms with distinct CN. b‐d) Reproduced with permission.^[^ ^107^ ^]^ Copyright 2024, Springer. e) AFM micrographs of cycled Zn electrodes using a CF separator. Reproduced with permission.^[^ ^115^ ^]^ Copyright 2024, Wiley‐VCH. f) In situ pH monitoring of ZnSO_4_ electrolyte over 10 h and corresponding cycling curves for Zn//Zn symmetric cells. Reproduced with permission.^[^ ^109^ ^]^ Copyright 2024, Wiley‐VCH. g) In situ electrochemical impedance spectroscopy (EIS) curves of Zn//Zn cells with ZnSO_4_ electrolyte. h) Tafel polarization plots for different electrolytes tested. g) and h) Reproduced with permission.^[^ ^116^ ^]^ Copyright 2025, Wiley‐VCH. i) Operando visualization of fractal zinc deposition under a 6 mA current (current density: 0.18 mA cm^−2^). Reproduced with permission.^[^ ^117^ ^]^ Copyright 2022, American Chemical Society.

The HER process in AZIBs follows a complex multi‐step mechanism. Initially, water undergoes slight ionization to generate H_3_O^+^. These hydronium ions migrate to the zinc anode surface, where they discharge to form atomic hydrogen (H^*^) and subsequently evolve H_2_. Although fortunately, the interaction between H^+^ and the Zn metal surface is very weak, resulting in a slow discharge step, which reduces the possibility of H_2_ evolution.^[^ ^99^ ^]^ Specifically, the Zn/Zn^2+^ redox potential (−0.76 V vs. SHE) lies below the H_2_O/H_2_ equilibrium (0 V), favoring preferential H^+^ reduction (**Figure** **5**a).^[^ ^42^, ^72^, ^100^ ^]^ However, AZIB still faces the challenge of a narrow electrochemical window (1.23 V) in aqueous electrolytes due to the decomposition of highly reactive H2O molecules, which promotes HER.^[^ ^101^ ^]^ Lin et al. demonstrated through Raman spectroscopy that during charging, [Zn(H_2_O)_6_]^2+^ migrates to the anode and undergoes desolvation, with elongated O─H bonds in coordinated water molecules readily dissociating to release free H^+^.^[^ ^62^ ^]^

Mechanistically, HER on zinc anode surfaces proceeds through two distinct steps: the Volmer step (proton adsorption, H^+^+e^−^+^*^→ H^*^) and the Tafel step (hydrogen desorption, 2H^*^ → H_2_).^[^ ^102^, ^103^ ^]^ First‐principles calculations have demonstrated that surface structure significantly influences the reaction kinetics. On atomically flat surfaces such as Zn (002) and (100), the Volmer step becomes rate‐determining, exhibiting high activation barriers (1.231 and 0.935 eV, respectively) and positive hydrogen adsorption free energies (Figure 5b). This energetics indicate weak hydrogen binding on flat surfaces. In contrast, uneven surfaces including Zn (101), (102), and (103) show lower Volmer barriers (0.684–0.759 eV) but higher Tafel barriers (0.938–0.965 eV), shifting the rate‐limiting step to the Tafel reaction (Figure 5c). The surface dependent HER activity correlates strongly with the generalized coordination numbers (CN) of surface zinc atoms. Flat surfaces, particularly Zn (002) with CN = 7.50, demonstrate reduced hydrogen adsorption strength. Conversely, uneven surfaces with lower coordination numbers (CN = 5.33–6.67) facilitate stronger hydrogen binding (Figure 5d). Surface energy calculations further confirm Zn (002) as the most thermodynamically stable facet.^[^ ^104^, ^105^, ^106^ ^]^ This fundamental relationship between surface coordination and HER kinetics suggests that promoting surface flatness could effectively suppress hydrogen evolution.^[^ ^107^ ^]^

To deepen the understanding of HER in AZIBs, both qualitative and quantitative analyses of hydrogen generation are essential. Electrochemical techniques such as linear sweep voltammetry (LSV) and Tafel analysis can assess HER intensity, while in situ optical microscopy enables direct visualization of hydrogen bubbles.^[^ ^108^ ^]^ Quantitative evaluation is achieved using in situ electrochemical gas chromatography, and pH monitoring can further verify HER occurrence.^[^ ^98^, ^109^ ^]^ Ultimately, HER is an unavoidable side reaction at the zinc anode due to water decomposition via chemical or electrochemical pathways. This leads to continuous consumption of active zinc and electrolyte, resulting in accelerated capacity fading and overall battery degradation.^[^ ^110^, ^111^, ^112^, ^113^ ^]^ The practical implications of HER are severe, as this parasitic reaction compromises CE and zinc utilization, while continuous gas evolution in sealed batteries leads to swelling and potential explosion risks.^[^ ^114^ ^]^

Closely coupled with HER, the corrosion process at the zinc/electrolyte interface represents another critical degradation mechanism. Zn metals experience very slow corrosion in neutral solutions. However, most aqueous electrolytes based on Zn salts are weakly acidic in practice (pH 3.6–6.0), which accelerates the corrosion process. The dissolution of Zn during cell rest (anodic process) is due to the presence of suitable electron acceptors in the electrolyte, such as protons, oxygen or water molecules. In a weakly acidic electrolyte, the corresponding cathodic process is dominated by the generation of H_2_ gas. the Zn atoms on the Zn surface enter the solution, and the Zn^2+^ and electrons migrate through the Zn metal to the cathodic reaction sites.^[^ ^118^, ^119^, ^120^, ^121^ ^]^ The primary corrosion reaction: Zn + 2H_2_O → Zn(OH)_2_ + H_2_↑is intrinsically linked to hydrogen evolution, where water decomposition not only generates H_2_ but also produces OH^−^ species that further accelerate zinc degradation. The corrosion mechanism is particularly sensitive to surface heterogeneity. Microscopic inhomogeneities create localized potential variations, forming numerous corrosion microcells. Through operando atomic force microscopy (AFM), Wong et al. revealed that these microcells preferentially initiate at high‐energy sites, similar to the preferential HER activity observed on uneven surfaces (Figure 5e).^[^ ^122^ ^]^ The generated OH‐ ions react with the anode surface, creating a self‐accelerating degradation cycle. This process is further complicated by the local pH evolution, as demonstrated by Hao et al. through systematic pH‐dependent studies. However, under weakly acidic conditions, this is accompanied by the precipitation of complex corrosion products (or layers) containing Zn. The accumulation of OH^−^ elevates interfacial pH, promoting the formation of insulating ZHS deposits (Figure 5f).^[^ ^109^ ^]^ These porous byproducts, when adhering to the anode surface, significantly impair electrode kinetics, with EIS revealing up to 300% resistance increase over 15 min (Figure 5g), and the corrosion rate is greatly accelerated (Figure 5h).^[^ ^116^ ^]^ The spatial distribution of corrosion sites, correlating strongly with electrode surface defects and crystal orientations, mirrors the surface‐dependent characteristics of HER.^[^ ^123^ ^]^ This parallel behavior suggests that surface engineering strategies might simultaneously address both degradation mechanisms.^[^ ^124^ ^]^

The formation of zinc dendrites, arising from the complex interplay of ion transport, solvation dynamics, and interfacial electric fields discussed in Section 2.1, represents the third major degradation phenomenon. Dendrites are branched, tree‐like metal deposits that grow perpendicular to the electrode surface, resulting from non‐uniform zinc deposition. Their formation is intrinsically linked to the local EDL structure and ion transport behavior at the electrode‐electrolyte interface.

The dendrite formation process follows a characteristic nucleation and growth mechanism. During initial zinc deposition, surface protrusions‐often created by HER‐induced damage or corrosion sites‐create localized electric field enhancements within the EDL.^[^ ^125^, ^126^, ^127^ ^]^ These enhanced fields disrupt the uniform [Zn(H_2_O)_6_]^2+^ transport and desolvation process, preferentially attracting Zn^2+^ to form nucleation sites. The nucleation kinetics follow Sand's time model (Equation (3)):

(3) $$\tau =\pi D\frac{eC_{0}{\left(\mu_{a}+\mu_{b}\right)}^{2}}{2J\mu_{a}}$$

where D, C_0_, J, and e are the diffusion coefficient, initial electrolyte concentration, effective current density, and electronic charge, respectively. μ_a_ and μ_b_ are the anion and cation mobility, respectively.^[^ ^115^, ^128^ ^]^ Therefore, the uniformity of the electric field is the main controlling factor for the presence of metallic dendrites.

Current density plays a critical role in zinc anode performance.^[^ ^129^ ^]^ At high current densities, the electric field becomes unevenly distributed, intensifying at dendritic tips and promoting localized deposition. In contrast, lower current densities result in a more uniform field.^[^ ^130^, ^131^ ^]^ Consequently, zinc electrodeposition is largely governed by the electric field's spatial distribution. According to classical nucleation theory, nucleation barriers affect the formation of atomic clusters.^[^ ^132^ ^]^ Even under low current densities, surface heterogeneities with varying nucleation energies lead to uneven zinc growth. Larger nucleation clusters at more active sites promote dendrite formation and potential intercalation.^[^ ^133^ ^]^ Subsequent crystal growth induces heterogeneous Zn^2+^ concentration gradients across the electrode surface, further amplifying electric field heterogeneity.^[^ ^9^ ^]^ The growth rate exhibits linear dependence on local current density, following the Cottrell relationship (Equation (4)):

(4) $$i=FAD^{1/2}\pi^{-1/2}t^{-1/2}$$

where i is the current, n is the number of electrons transferred, F is the Faraday constant, A is the electrode area, D is the diffusion coefficient, and t is time.^[^ ^134^ ^]^ This relationship highlights how ion transport limitations, particularly at high current densities, accelerate dendrite propagation. Sand's time model has systematically demonstrated how operating conditions (current density, temperature, and electrolyte composition) affect dendrite morphology through their influence on local ion transport and EDL structure.^[^ ^135^, ^136^ ^]^ The morphological evolution of dendrites, as revealed by in situ operando digital microscopy, shows distinct transitions from initial nuclei to branched structures (Figure 5i).^[^ ^117^ ^]^ This evolution creates a self‐reinforcing cycle: dendrite growth sites alter local electric fields and ion transport pathways, which in turn promote further non‐uniform deposition. Moreover, these dendrite sites often become preferential locations for HER and corrosion, due to their high surface energy and disrupted EDL structure, creating a complex interplay between all three degradation mechanisms.^[^ ^137^ ^]^

These three critical issues exhibit complex interactions that form self‐accelerating feedback loops, as demonstrated through multi‐modal characterization techniques.^[^ ^138^ ^]^ The HER‐generated OH^−^ promotes local corrosion, while corrosion products increase surface roughness, providing more HER active sites. Both processes contribute to zinc loss and gas accumulation. Surface irregularities from corrosion provide preferential nucleation sites for dendrites, while the uneven distribution of corrosion products leads to localized current density concentration.^[^ ^139^, ^140^ ^]^ The high curvature at dendrite tips further accelerates local corrosion.^[^ ^141^ ^]^ Dendrites increase the effective surface area for HER, while HER‐induced bubbles significantly disturb electrolyte ion distribution. These issues form positive feedback loops, mutually accelerating each other. The exacerbation of any single issue promotes the deterioration of others, resulting in rapid battery performance decay. Therefore, effective protection strategies must address these synergistic effects rather than targeting individual issues in isolation.^[^ ^142^ ^]^ This explains why single protection methods often show limited effectiveness, necessitating the development of multifunctional protective approaches.^[^ ^143^, ^144^ ^]^

## 3 Structure and Function of Biomass‐Derived Functional Groups

Functional groups serve as the fundamental molecular units that determine the chemical and electrochemical properties of biomass‐derived materials in AZIBs. Through their distinct electronic configurations and bonding interactions, these groups regulate critical battery processes including ion transport kinetics, interfacial reactions, and zinc deposition behavior. The strategic modification of functional groups has demonstrated significant improvements in key AZIB performance metrics such as energy density, cycling stability and CE.

This section systematically analyzes how different functional groups in biomass materials influence AZIB performance through three aspects: 1) the intrinsic chemical structure and properties of each functional group, 2) their specific roles and mechanisms in regulating battery processes based on reported studies, and 3) their synergistic effects when integrated into biomass materials. This structure‐function analysis aims to provide clear design principles for developing high‐performance biomass‐based materials for AZIBs.

### 3.1 Carboxyl Groups

**Figure 6:**
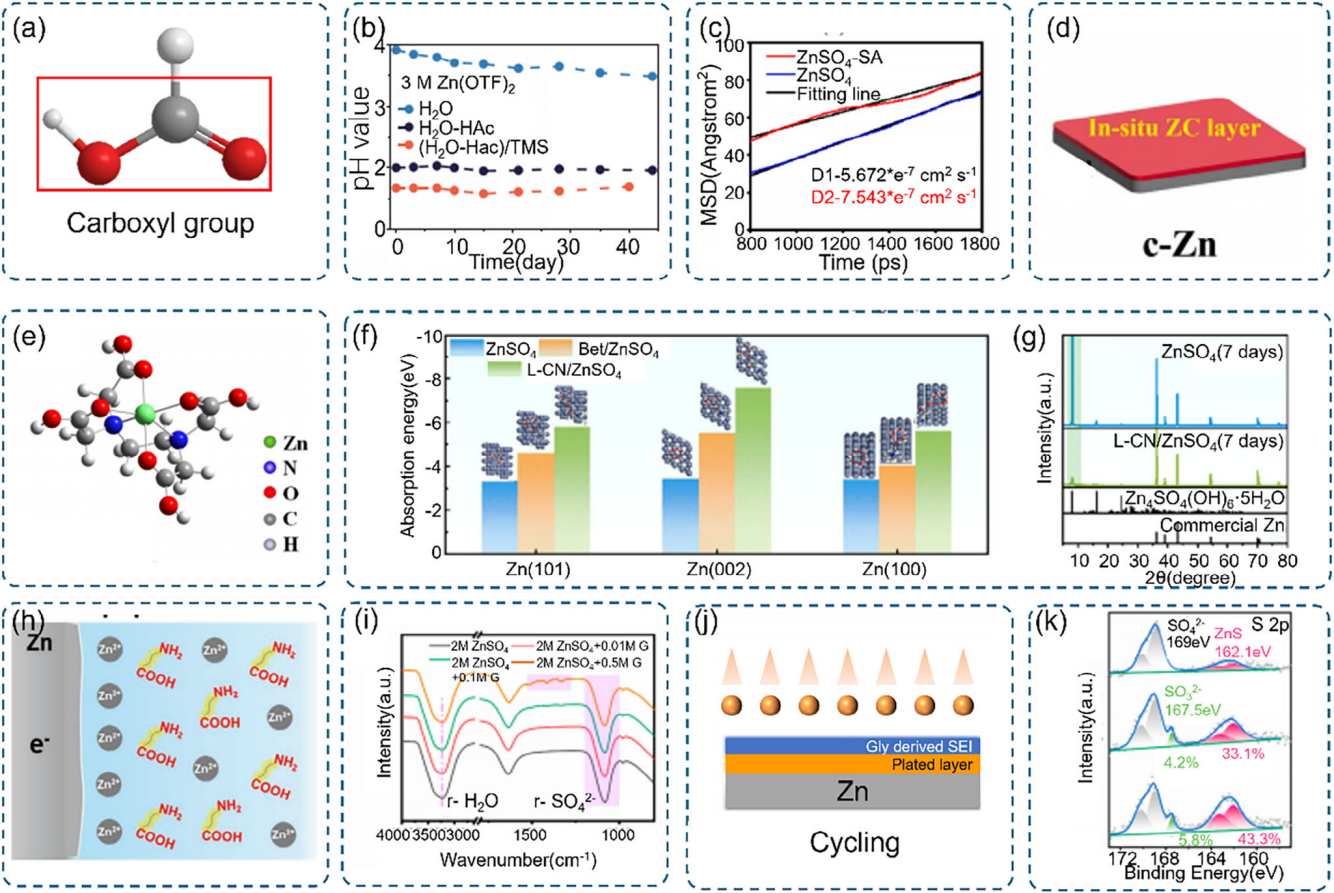
a) Schematic representation of the carboxyl functional group. b) Variation in pH values over time for electrolytes consisting of 3 m Zn(OTF)_2_ dissolved in pure H_2_O, H_2_O‐HAc, and (H_2_O‐HAc)/TMS solutions, respectively. Reproduced with permission.^[^ ^149^ ^]^ Copyright 2022, Wiley‐VCH. c) Mean squared displacement (MSD) profiles in ZnSO_4_ and ZnSO_4_‐SA electrolytes. Reproduced with permission.^[^ ^150^ ^]^ Copyright 2023, Elsevier. d) Schematic diagram of the synthetic approach for preparing the c‐Zn electrode using a CA solution. Reproduced with permission.^[^ ^151^ ^]^ Copyright 2024, Elsevier. e) Molecular structure of the [ZnH_4_EDTA]^2+^ complex cation. Reproduced with permission.^[^ ^152^ ^]^ Copyright 2022, American Chemical Society. f) Energy comparison of hydrated [(Zn(H_2_O)_6_]^2+^, [(Zn(H_2_O)_5_(Bet)]^2+^, and [(Zn(H_2_O)_5_(L‐CN)]^2+^ species adsorbed on the Zn (002) crystal plane. g) X‐ray diffraction (XRD) patterns of Zn foil after immersion in electrolytes with and without the L‐CN additive for 7 days. f) and g) Reproduced with permission.^[^ ^153^ ^]^ Copyright 2023, Royal Society of Chemistry. h) Schematic illustration of the strategy for suppressing Zn dendrite formation in ZnSO_4_ electrolytes containing silk peptide (SP) additives. Reproduced with permission.^[^ ^154^ ^]^ Copyright 2022, Wiley‐VCH. i) Fourier‐transform infrared (FT‐IR) spectra of 2 m ZnSO_4_ electrolyte without and with different concentrations of Glycine (Gly). j) Schematic diagram of the synthetic method for fabricating the c‐Zn electrode using a Gly solution. k) High‐resolution S_2p_ X‐ray photoelectron spectroscopy (XPS) depth spectra of the Zn anode after 20 cycles in Gly/ZnSO_4_ electrolyte. i)‐k) Reproduced with permission.^[^ ^155^ ^]^ Copyright 2023, American Chemical Society.

Recent studies have demonstrated that the carboxyl group (─COOH) plays a crucial role in AZIBs due to its unique molecular structure and electronic properties.^[^ ^145^, ^146^ ^]^ The fundamental structure of carboxyl groups consists of a carbonyl moiety (C═O) connected to a hydroxyl group (─OH) (**Figure** **6**a). Research has revealed that the strong electronegativity of oxygen atoms in both components significantly influences the electronic distribution within the group. The oxygen atom in the carbonyl group draws electron density from the carbon–oxygen double bond, generating a partial positive charge on the carbon atom. Similarly, the hydroxyl oxygen polarizes the O─H bond, facilitating proton (H^+^) dissociation and contributing to the acidic nature of carboxyl groups. Studies have further established that the electronic structure of carboxyl groups is stabilized through p‐π conjugation between the carbonyl π‐bond and the hydroxyl oxygen lone pair electrons. This conjugation mechanism leads to more uniform electron distribution and enhanced group stability while strengthening the acidic character beyond that of typical alcohol hydroxyl groups.^[^ ^147^, ^148^ ^]^ These fundamental structural characteristics and electronic properties make carboxyl groups particularly effective in modulating various aspects of AZIB performance.^[^ ^146^ ^]^

The acidic nature of carboxyl groups, arising from their partial proton dissociation capability, enables effective pH regulation in AZIBs. This proton exchange capacity maintains electrolyte stability by counteracting alkaline by‐products generated from zinc anode side reactions. Beyond pH control, the proton exchange mechanism significantly influences zinc ion transport processes, affecting both migration and diffusion kinetics throughout the battery system.

Several experimental studies have demonstrated these regulatory effects. Pan and colleagues employed acetic acid (HA_C_) as a buffer additive, establishing stable pH conditions that effectively suppressed OH‐induced precipitation at zinc electrodes (Figure 6b).^[^ ^149^ ^]^ In a separate investigation, Fan and coworkers revealed that succinic acid (SA) addition markedly enhanced zinc ion transport, increasing the Zn^2+^ diffusion coefficient from 5.672 to 7.543 cm^2^ s^−1^ as measured by mean square displacement analysis (Figure 6c).^[^ ^150^ ^]^ Carboxyl groups in SA increase Zn^2+^‐SO_4_^2−^ contact ion pairs, reducing water‐mediated side reactions. Their work uncovered a strong coupling between carboxylate proton dissociation equilibrium and zinc ion insertion‐extraction processes. During zinc ion insertion, dissociated protons rapidly migrate to the counter electrode through the electrolyte, maintaining charge balance and consequently enhancing overall battery charging–discharging kinetics.

Strong interfacial interactions between carboxyl groups and zinc metal enable the formation of protective surface films through preferential adsorption and chemical reactions. Shi and colleagues demonstrated that citric acid treatment generates protective zinc citrate layers on zinc electrodes (Figure 6d).^[^ ^151^ ^]^ Similarly, Fan and coworkers reported the formation of an in situ passivation layer (‐C_5_H_4_O_4_‐Zn‐O_4_H_4_C_5_‐) through co‐reduction reactions between itaconic acid and zinc.^[^ ^156^ ^]^ These stable protective layers effectively isolate reactive water molecules from the zinc surface, suppressing side reactions while maintaining uniform zinc ion transport.

Beyond surface protection, carboxyl groups demonstrate remarkable coordination chemistry with zinc ions through their carbonyl and hydroxyl oxygen atoms. The lone electron pairs of these oxygen atoms form stable coordination bonds, enabling precise regulation of zinc ion solvation structures in aqueous environments. Wang and colleagues illustrated this effect using EDTA additives, which form [Zn(H_4_Y)]^2+^ complexes (Figure 6e). These coordination complexes modify the zinc ion solvation environment by reducing water molecule content in the solvation shell, thereby suppressing HER and facilitating zinc ion insertion processes.^[^ ^152^ ^]^

Carboxyl groups, abundantly present in biomass materials like amino acids and proteins, demonstrate diverse regulatory functions in AZIBs. Chen and colleagues investigated L‐carnitine (L‐CN), where carboxyl groups facilitate protective film formation through zinc ion binding. Their density functional theory calculations revealed that [Zn(H_2_O)_5_(L‐CN)]^2+^ complexes exhibit stronger binding energies on zinc (002) surfaces compared to [Zn(H_2_O)_6_]^2+^, promoting preferential zinc deposition (Figure 6f), this is consistent with the FTIR results(Figure S1, Supporting Information). Carboxyl groups in L‐CN show potential‐dependent binding energy changes, adjusting the protective layer thickness during charging/discharging. This adaptability enhances Zn^2+^ desolvation kinetics. XRD analysis confirmed this theoretical prediction, showing selective zinc deposition along the (002) plane that enhanced stripping/plating stability (Figure 6g).^[^ ^153^ ^]^

Additional biomass‐derived systems further demonstrate carboxyl group functionality. Wang and coworkers explored SP, finding that shorter peptide chains expose more carboxyl groups for enhanced zinc ion chelation and stable adsorption layer formation (Figure 6h).^[^ ^154^ ^]^ This mechanism effectively stabilizes zinc anodes by controlling zinc ion deposition and suppressing dendrite growth. Mai and colleagues examined glycine additives, revealing strong zinc ion binding that modifies solvation structures through [Zn(H_2_O)_5_Gly]^2+^ complex formation (Figure 6i). This modification reduces SO_4_^2−^ contact ion‐pair ratios and suppresses side reactions. Moreover, glycine's carboxyl groups form a dynamic adsorption layer that evolves into an ionically conductive (Figure 6j), chemically stable ZnS‐rich SEI through reduction processes (Figure 6k).^[^ ^155^ ^]^

### 3.2 Amino Group

**Figure 7:**
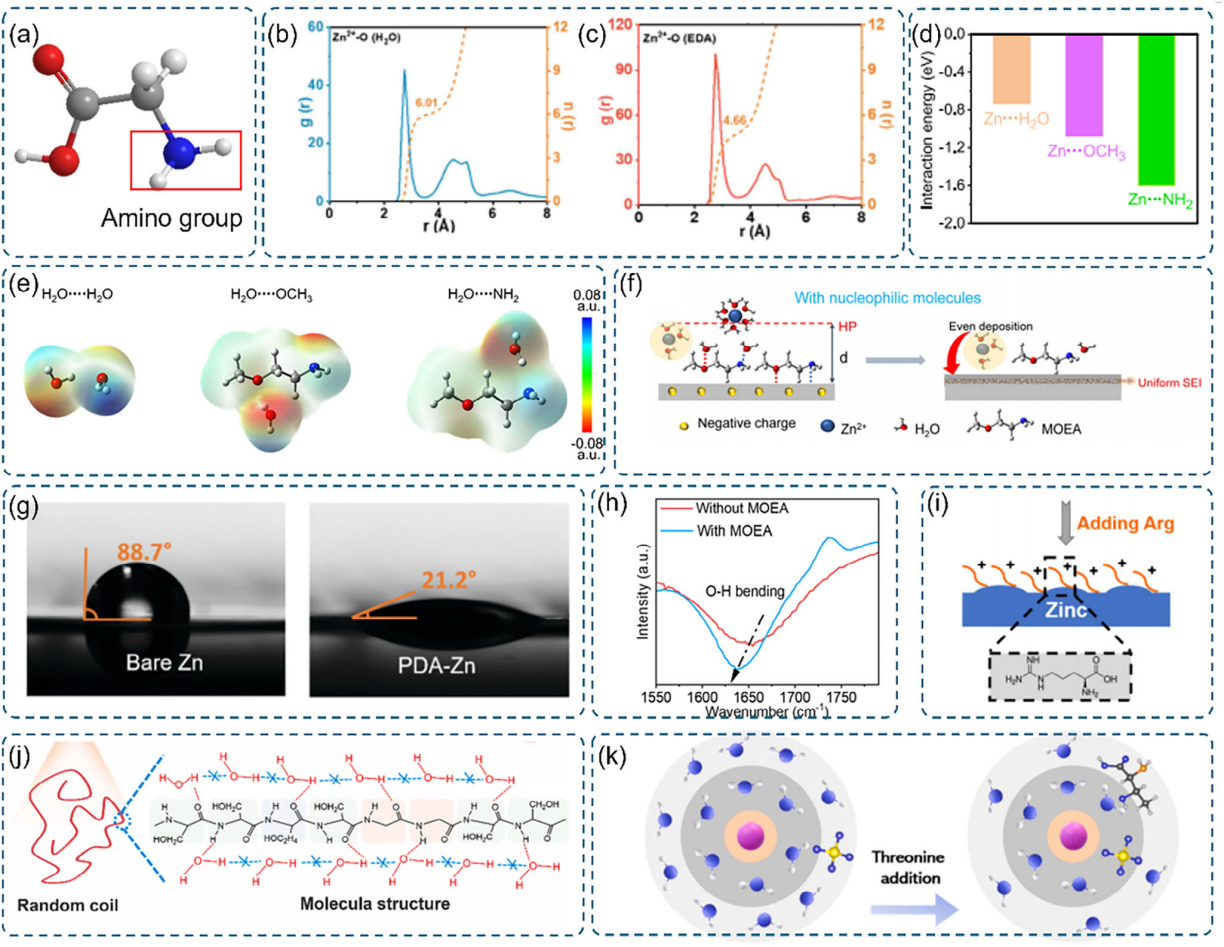
a) Schematic representation of the amino functional group. b) Radial distribution function (RDF) of the ZnSO_4_ solution. c) Radial distribution function (RDF) of the ZnSO_4_ + EDA solution. b) and c) Reproduced with permission.^[^ ^159^ ^]^ Copyright 2024, Applied Physics Letters. d) DFT‐calculated adsorption energies of H_2_O and MOEA molecules on the Zn anode surface. e) Charge distribution diagrams for hydrogen‐bonded molecular pairs: H_2_O∙∙∙H_2_O, H_2_O∙∙∙OCH_3_, and H_2_O∙∙∙NH_2_. f) Schematic diagram of the HP (presumably a protective layer) and the Zn deposition process on the anode. g) Contact angle measurements of the electrolyte on a bare Zn electrode versus a PDA–Zn electrode. Reproduced with permission.^[^ ^160^ ^]^ Copyright 2021, Royal Society of Chemistry. h) FT‐IR spectra of electrolytes with and without the MOEA additive in the 1550–1800 cm^−1^ wavenumber range. d)‐f) and h) Reproduced with permission.^[^ ^161^ ^]^ Copyright 2025, American Chemical Society. i) Schematic illustration of Zn electrodeposition behavior in the presence of the Arginine (Arg) additive. Reproduced with permission.^[^ ^162^ ^]^ Copyright 2021, Wiley‐VCH. j) Diagram depicting the layer structure, secondary structure of sericin, and disruption of the hydrogen bond network between water molecules. Reproduced with permission.^[^ ^163^ ^]^ Copyright 2025, Elsevier. k) Schematic showing changes in the solvation structure of Zn^2+^ ions after adding TH to the electrolyte. Reproduced with permission.^[^ ^16^ ^]^ Copyright 2022, Elsevier.

The amino group (─NH_2_) features a central nitrogen atom covalently bonded to two hydrogen atoms in a specific spatial arrangement (**Figure** **7**a). This nitrogen atom possesses a lone electron pair, fundamentally determining the chemical properties of amino‐containing compounds. The electron‐donating capability of this lone pair enables diverse chemical interactions, particularly the formation of coordination bonds with electron‐deficient species. Additionally, the nitrogen atom's electronegativity, enhanced by its lone electron pair, influences local electron density distributions during molecular interactions. Such electronic effects manifest notably in organic compounds, where amino groups modify the electron density of adjacent carbon atoms, thereby altering molecular reactivity and chemical properties.^[^ ^157^, ^158^ ^]^

Amino groups exhibit strong electrostatic interactions with zinc ions (Zn^2+^), forming stable complexes through coordination bonds. The nitrogen atom's lone electron pair fills zinc's empty orbitals, establishing stable coordination structures that regulate zinc ion transport in electrolytes. This coordination mechanism enhances zinc ion migration uniformity at electrode/electrolyte interfaces. For example, ethylenediamine (EDA) addition to ZnSO_4_ electrolytes modifies zinc ion solvation structures, reducing active water involvement while promoting uniform zinc deposition and suppressing dendrite growth, thereby improving battery cycling stability and safety (Figure 7b,c).^[^ ^159^ ^]^

The preferential binding characteristics of amino groups with zinc surfaces have been quantitatively demonstrated through computational studies. Xu and colleagues employed density functional theory calculations to investigate methoxy ethylenediamine (MOEA) interactions with zinc anodes. Their results revealed stronger adsorption energies for MOEA's amino (−1.60 eV) and methoxy (−1.07 eV) groups compared to water molecules (−0.73 eV) (Figure 7d,e). In the common electrolyte, the peak positions are located at 531.9 and 530.1 eV, which can be attributed to ─OH and Zn─O, possibly corresponding to the formation of Zn_4_SO_4_(OH)_6_‐H_2_O and Zno. Meanwhile, in the electrolyte with MOEA, the peak positions are located at 531.5 and 530.1 eV, which can be attributed to the adsorbed MOEA and Zn─O, C─O(Figure S2, Supporting Information). This preferential binding facilitates the formation of stable solid electrolyte interphase layers while suppressing side reactions (Figure 7f).^[^ ^161^ ^]^

Amino groups enhance electrode‐electrolyte interactions through improved hydrophilicity in AZIBs (Figure 7g).^[^ ^160^ ^]^ The formation of hydrogen bonds between electronegative amino groups and water molecules increases electrolyte affinity for electrode materials, promoting efficient ion transport and improving battery performance (Figure 7h). This wettability enhancement particularly benefits the electrode‐electrolyte interface, where efficient ion transport critically determines charging–discharging capabilities.

In biomass materials, amino groups function through synergistic interactions with neighboring functional groups. Jiang and colleagues demonstrated this synergy using Arg molecules, where electrostatic interactions enable preferential absorption on zinc surfaces (Figure 7i), simultaneously suppressing water adsorption and hydrogen evolution while promoting uniform zinc deposition.^[^ ^162^ ^]^ Hu and coworkers explored Silk Serine (SS) as an electrolyte additive (Figure 7j), utilizing its unique structural characteristics. Amino groups in SS enable facile dissolution in mild acids, facilitating electrode recycling without harsh chemicals. The macromolecular bioprotein's layered structure and random coil conformation expose multiple polar groups, facilitating extensive hydrogen bonding networks.^[^ ^163^ ^]^ Wang and colleagues further revealed threonine's role in modifying zinc ion solvation environments. Their studies showed that threonine molecules weaken H_2_O‐Zn^2+^ interactions while strengthening SO_4_^2−^‐Zn^2+^ coordination, fundamentally altering solvation sheath structures (Figure 7k).^[^ ^16^ ^]^

### 3.3 Hydroxyl Group

**Figure 8:**
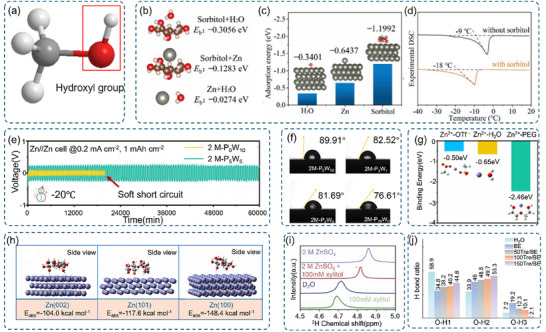
a) Schematic representation of the hydroxyl functional group. b) Visual illustration and associated binding energy values for different molecular/ionic couples. c) Adsorption energy values of H_2_O molecules, Zn atoms, and sorbitol molecules onto the Zn (002) crystal surface. d) Differential scanning calorimetry (DSC) curve comparison between electrolytes without sorbitol and with 10% sorbitol. b)‐d) Reproduced with permission.^[^ ^168^ ^]^ Copyright 2023, Elsevier. e) Voltage profile evolution of Zn//Zn symmetric cells in various electrolytes at −20 °C. f) Contact angle measurements of a series of cosolvent electrolytes on a Zn electrode at 20 °C. g) Relative binding energies of Zn^2+^ with H_2_O, PEG, and OTf^−^, derived from DFT calculations. e)‐g) Reproduced with permission.^[^ ^169^ ^]^ Copyright 2022, Elsevier. h) Adsorption energies of Malt molecules on Zn (002), (100), and (101) crystal planes. Reproduced with permission.^[^ ^170^ ^]^ Copyright 2024, Elsevier. i) ^2^H NMR spectra of H_2_O molecules in pure D_2_O, 2 m ZnSO_4_, 100 mm xylitol, and 2 m ZnSO_4_ containing 100 mm xylitol. Reproduced with permission.^[^ ^171^ ^]^ Copyright 2023, Wiley‐VCH. j) Summary of correlations between the ratio of hydrogen bonds and observed phenomena. Reproduced with permission.^[^ ^66^ ^]^ Copyright 2024, Wiley‐VCH.

The hydroxyl group (─OH) consists of a covalently bonded oxygen and hydrogen atom, with its chemical properties largely determined by oxygen's high electronegativity (**Figure** **8**a). In the covalent bond, the shared electron pair strongly biases toward oxygen, creating partial negative and positive charges on the oxygen and hydrogen atoms respectively, resulting in significant molecular polarity. This polarity enables crucial interactions with other charged or polar species, particularly through hydrogen bonding. Spatially, the oxygen atom exhibits sp^3^ hybridization, where two hybrid orbitals form σ‐bonds (one with hydrogen and potentially another with adjacent atoms in organic compounds), while the remaining two hybrid orbitals contain lone electron pairs. This electronic configuration and spatial arrangement confers specific molecular orientations and steric effects that influence broader molecular properties including solubility and intermolecular interactions.^[^ ^164^, ^165^, ^166^ ^]^

**Figure 9:**
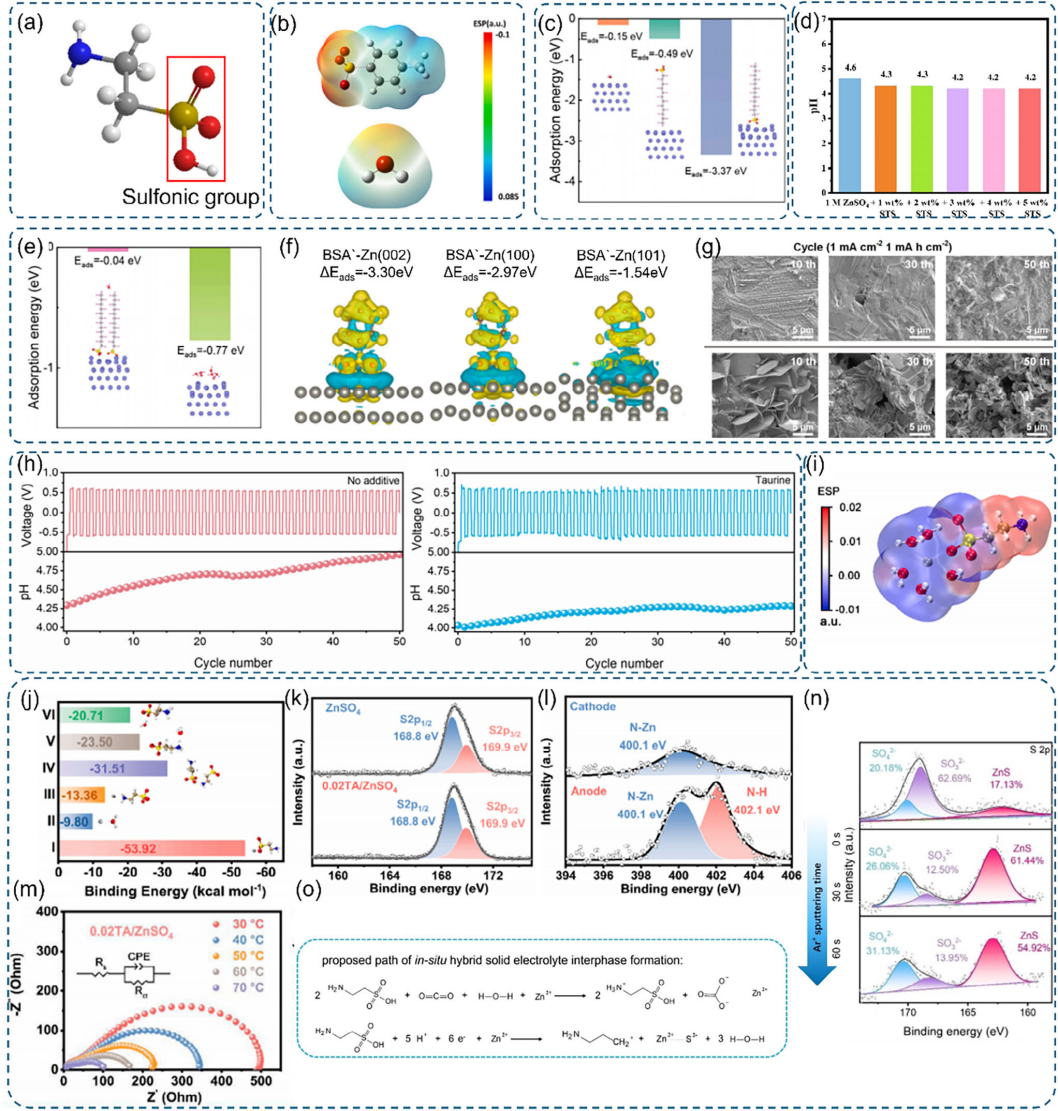
a) Schematic illustration of the sulfonic acid functional group. b) Electrostatic potential (ESP) maps of the TS^−^ ion and H₂O molecule. c) Adsorption energies of water molecules and SDS on Zn substrates. d) pH values of the ZnSO_4_ electrolyte with varying concentrations of STS. b) and d) Reproduced with permission.^[^ ^178^ ^]^ Copyright 2024, Elsevier. e) Adsorption energies for the Zn substrate/water molecule and Zn substrate/SDS molecular layer on water molecule interactions. c) and e) Reproduced with permission.^[^ ^179^ ^]^ Copyright 2024, Elsevier. f) Comparison of BSA adsorption energies on different Zn crystal planes and the corresponding charge density differences. g) Scanning Electron Microscope (SEM) images of Zn foils after 1 h electrodeposition and after various cycles in SBS/ZnSO_4_ and pure ZnSO_4_ electrolytes using symmetric cells. f) and g) Reproduced with permission.^[^ ^180^ ^]^ Copyright 2024, Elsevier. h) In situ pH monitoring of 2 m ZnSO_4_ and 2 m ZnSO_4_ + 50 m m taurine in Zn//Zn symmetric cells during cycling at 20 mA cm^−2^ with a capacity of 5 mAh cm^−2^. i) ESP distribution within the solvation structure. j) Binding energies of Zn‐SO_3_ (I), Zn‐H_2_O (II), Zn‐NH_3_ (III), NH_3_‐SO_3_ (IV), NH_3_‐H_3_O (V), and SO_3_‐H_3_O (VI) pairs. k) Corresponding high‐resolution S_2p_ XPS spectra. l) High‐resolution N_1s_ XPS spectra of Zn anode and cathode after 100 cycles in 0.02TA/ZnSO_4_ electrolyte. m) Nyquist plots of cells tested at different temperatures using 0.02TA/ZnSO_4_ electrolyte. l)‐m) Reproduced with permission.^[^ ^181^ ^]^ Copyright 2023, Elsevier. n) XPS depth profiles for S_2p_ on the Zn anode surface in electrolytes with and without taurine after 50 cycles, along with the proposed in situ interface formation pathway. o) Proposed mechanism for the in situ formation of a hybrid solid electrolyte interphase. h), n), and o) Reproduced with permission.^[^ ^182^ ^]^ Copyright 2023, Wiley‐VCH.

Hydroxyl‐containing additives demonstrate remarkable low‐temperature tolerance through hydrogen bonding interactions with water molecules.^[^ ^167^ ^]^ Sorbitol's multiple hydroxyl groups disrupt water's regular ice crystal structure by redistributing hydrogen bonds (Figures 8b and **9**c), reducing electrolyte freezing points from −9 to −18 °C at 10% concentration (Figure 8d).^[^ ^168^ ^]^ Hydroxyl groups in sorbitol disrupt the H_2_O‐H_2_O network, reducing free water activity and shifting the Zn^2+^ solvation shell from [Zn(H_2_O)_6_]^2+^ to [Zn(H_2_O)_5_(OH)]^+^. Similarly, polyethylene glycol (PEG) molecules alter water's hydrogen bonding network and decrease free water activity, achieving freezing point depression to −93.04 °C in certain formulations. The battery can be stably cycled for 1000 h at a low temperature of −20 °C (Figure 8e).^[^ ^169^ ^]^

Beyond freezing point depression, hydroxyl groups enhance electrode‐electrolyte compatibility through improved wettability and interface modification. PEG addition reduces electrolyte‐electrode contact angles (Figure 8f), ensuring better zinc anode wetting and maintaining efficient ion transfer at low temperatures. Polydopamine (PDA) and similar hydroxyl‐rich compounds form hydrophilic, ionically conductive solid electrolyte interface layers through strong zinc ion interactions. These surface modifications promote uniform zinc deposition while maintaining electrode stability at low temperatures, effectively suppressing side reactions and enhancing overall battery performance in cold conditions.^[^ ^160^ ^]^

Hydroxyl groups significantly influence zinc ion solvation structures in aqueous electrolytes through competitive coordination mechanisms. When introduced into electrolytes, hydroxyl‐containing compounds like PEG compete with water molecules for zinc ion coordination, modifying the solvation environment and reducing hydrated zinc ion populations (Figure 8g). These structural modifications enhance zinc ion transport and overall electrochemical performance.^[^ ^168^ ^]^

Biomass materials rich in hydroxyl groups demonstrate particularly effective zinc ion regulation. Compounds such as glucose, sucrose (Suc), xylitol, and trehalose exhibit lower zinc ion binding energies compared to water molecules, promoting preferential zinc coordination as confirmed by NMR studies.^[^ ^66^, ^171^, ^172^, ^173^ ^]^ This preferential binding reduces reactive water molecule concentrations near zinc surfaces, suppressing side reactions. For instance, maltose (Malt) modifies interfacial chemistry by establishing intermolecular hydrogen bonds and dynamic Helmholtz planes, promoting uniform zinc deposition along (002) crystal planes while reducing water and sulfate concentrations (Figure 8j).^[^ ^170^ ^]^ Hu's research demonstrated xylitol's ability to decrease free water activity, evidenced by NMR chemical shift changes from 4.715 to 4.690 ppm at 100 m m concentration (Figure 8h).^[^ ^171^ ^]^ Similarly, Fan's studies with trehalose showed expanding hydrogen bonding networks through hydroxyl group electron donation to water molecules, indicated by increasing O‐H2 ratios (Figure 8l).^[^ ^66^ ^]^

In addition, we summarize the relationship between the density of hydroxyl functional groups and ionic conductivity and interfacial resistance as described above (Table S3, Supporting Information). Glucose (50 mm) had the highest ionic conductivity (129 mS cm^−1^) despite its low functional group density, which may be related to the facilitation of ionic migration by its molecular structure. Xylitol (500 mm) had the highest functional group density, but the ionic conductivity was only 75 mS cm^−1^, suggesting that the high functional group density may inhibit ionic migration due to enhanced hydrogen bonding. The conductivity of alginate (80 mm) and α‐CD (180 mm) decreased with the increase of functional group density, which further indicated that the relationship between functional group density and conductivity was not simply positive, but might be affected by the molecular configuration. Xylitol (500 mm) had the lowest interfacial resistance (54 Ω), possibly due to its high functional group density which promotes compatibility of the electrolyte with the interface. Trehalose (80 mm) had the highest interfacial resistance (500 Ω), presumably due to its low functional group density resulting in poor interfacial stability. Maltose (40 mm) and α‐ cyclodextrin (180 mm) had the same interfacial resistance (300 Ω), suggesting that there may be a threshold for the effect of functional group density on interfacial resistance in this interval. In conclusion, the effect of functional group density on ionic conductivity and interfacial resistance is complex and not a single linear relationship. Glucose exhibits optimal ion transport properties at low functional group densities, while xylitol achieves the lowest interfacial resistance at high functional group densities. This suggests that the synergistic effect of functional group density and molecular structure on the electrochemical properties should be considered in the material design, rather than focusing on the modulation of a single parameter.

### 3.4 Sulfonic Acid Group

The sulfonic acid group (─SO_3_H) features a central sulfur atom bonded to three oxygen atoms in distinct configurations, with one oxygen forming a hydroxyl group (─OH) while the sulfur also bonds to carbon or other atoms (Figure 9a). This structural arrangement creates unique electronic properties through multiple bonding interactions.^[^ ^174^ ^]^ The high electronegativity of oxygen atoms significantly influences electron distribution within sulfonic acid groups. Both sulfur–oxygen double bonds (S═O) and single bonds (S─O) exhibit strong electron density bias toward oxygen, creating partial positive charge on sulfur and enhancing its electron‐withdrawing capacity. The hydroxyl component's oxygen similarly draws electron density from hydrogen, facilitating H^+^ dissociation and contributing to the group's acidic character in aqueous solutions. Furthermore, sulfonate groups establish p‐π conjugated systems that enable electron delocalization. This conjugation enhances group stability while strengthening acidic properties by efficiently dispersing negative charge across the sulfonate ion (‐SO_3_‐), promoting H^+^ dissociation. The combined electronic effects of electronegativity and conjugation fundamentally determine the group's chemical reactivity and stability.^[^ ^175^, ^176^, ^177^ ^]^

The sulfonic acid group demonstrates exceptional capability in optimizing zinc ion solvation structures through strong zincophilic properties. Zhou and colleagues' research with sodium p‐toluene sulfonate (STS) revealed that the sulfonic acid group's interaction with zinc ions (Zn^2+^) significantly exceeds water molecule attraction (Figure 9b). This preferential interaction enables toluenesulfonate ions (TS^−^) to effectively displace water molecules from zinc ion solvation shells, reducing active water molecule concentrations around zinc centers. This solvation structure modification proves crucial for battery performance enhancement by suppressing water decomposition during zinc ion deposition, thereby reducing hydrogen evolution and other detrimental side reactions. Nuclear magnetic resonance studies demonstrated progressive ^2^H peak shifts with increasing STS concentration, confirming enhanced TS^−^ participation in zinc ion solvation shells and reduced Zn^2+^‐H_2_O coordination. This experimental evidence directly correlates sulfonic acid group presence with improved electrochemical stability through solvation structure optimization.^[^ ^178^ ^]^

The sulfonic acid group is also excellent in inhibiting side reactions, which can be achieved through multiple mechanisms. It exhibits strong adsorption on zinc surfaces, forming protective layers (Figure 9c) that act as barriers against water molecules, significantly reducing potential side reactions at zinc anode surfaces. Sulfonate groups in SDS adsorb rapidly but desorb slowly, forming a “molecular anchor” that stabilizes the SEI layer during volume changes. Sodium dodecyl sulfonate (SDS) exemplifies this functionality, where its sulfonic acid group's surface adsorption combines synergistically with its hydrophobic alkyl chain to restrict free water access, effectively suppressing HER and corrosion.^[^ ^179^ ^]^ Additionally, sulfonic acid‐containing additives demonstrate pH regulation capabilities that inhibit alkaline by‐product formation. For instance, STS addition decreases electrolyte pH (Figure 9d), effectively preventing the formation of alkaline interfacial products like ZHS and consequently enhancing zinc anode cycling stability.

The sulfonic acid group plays a crucial role in regulating zinc deposition behavior through multiple mechanisms. In SDS systems, strong electrostatic attractions between sulfonic acid groups and zinc ions create localized high‐concentration layers at anode surfaces, promoting 3D zinc ion diffusion (Figure 9e). This enhanced 3D distribution ensures more uniform zinc ion deposition, effectively counteracting dendrite formation caused by uneven deposition patterns. Similarly, sodium benzene sulfonate (SBS) demonstrates selective crystal plane interactions, where BSA^−^ preferentially adsorbs on Zn(002) planes (Figure 9f). This selective adsorption forms protective layers that moderate Zn^2+^ deposition rates on (002) planes, redirecting deposition to alternative crystal planes and promoting columnar (002) diffractive crystal growth while suppressing dendrite formation. Scanning electron microscopy evidence (Figure 9g) confirms these effects, revealing denser, more uniform zinc deposition layers with refined grain structures following SBS addition, demonstrating the sulfonic acid group's effectiveness in controlling zinc deposition morphology.^[^ ^180^ ^]^

**Figure 10:**
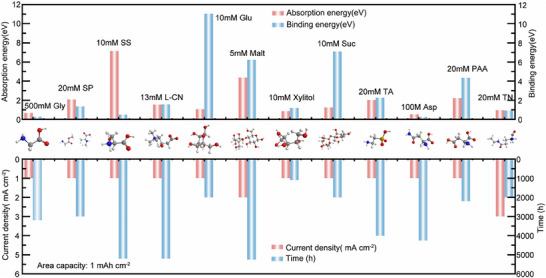
Comparison chart of the addition amount, adsorption energy, binding energy, cycle conditions and cycle life of some biomass materials.

Taurine's sulfonic acid groups enhance zinc anode stability in AZIBs through three synergistic mechanisms. First, they provide pH buffering capabilities by releasing H^+^ ions to neutralize HER‐produced OH^−^, preventing ZHS by‐product formation (Figure 9h). ─SO_3_H releases H^+^ to neutralize OH^−^ from HER, while ─NH_2_ buffers excess H^+^. This dynamic buffering surpasses static pH control, enabling self‐healing of the interface. Second, sulfonic acid groups modify zinc ion solvation structures through direct coordination. Molecular dynamics simulations reveal taurine molecules displacing water in primary solvation shells, forming [Zn(H_2_O)_5_TA]^2+^ complexes that reduce reactive water content and suppress HER (Figure 9i). Third, strong interfacial engineering occurs through substantial adsorption (−53.92 kcal mol^−1^ binding energy) (Figure 9j), creating hydrophobic bilayers that restrict water access. Spectroscopic evidence (XPS and Raman) confirms the formation of dynamic adsorption layers containing both sulfonic acid and amino groups that inhibit dendrite growth (Figure 9k,l). The bifunctional nature of taurine enables enhanced synergy, where ─SO_3_H and ─NH_2_ form dynamic salt bridges facilitating directional zinc ion transport, demonstrated by reduced interfacial resistance in EIS analysis (Figure 9m). Furthermore, electrochemical reduction generates a ZnS‐organic hybrid interface that isolates zinc from water, with XPS depth analysis revealing ZnS enrichment accelerating desolvation kinetics (Figure 10n,o).^[^ ^181^, ^182^ ^]^

## 4 Comparison and Discussion

Through systematic analysis of reported biomass‐derived materials for zinc protection (**Figure** **10**).^[^ ^70^, ^153^, ^154^, ^155^, ^163^, ^170^, ^171^, ^172^, ^173^, ^181^, ^183^, ^184^ ^]^ we have identified several critical structure‐function relationships that provide new insights into interface engineering strategies. The comprehensive comparison of binding energies, adsorption characteristics, and cycling performance reveals intriguing patterns in how molecular structures influence protective mechanisms.

### 4.1 Fundamental Structure‐Performance Relationships of Functional Groups

Our analysis of the binding energy data shows a consistent hierarchy among the functional groups. The binding energies vary significantly among different molecules, with Glu showing the highest binding energy (≈11 eV), followed by SS and Suc (both ≈7–8 eV).^[^ ^163^, ^172^, ^173^ ^]^ Most other molecules exhibit moderate binding energies in the range of 1–3 eV. This suggests that binding energy is more related to the overall molecular structure rather than simply the type of functional group. Interestingly, this binding energy trend does not directly translate into linear ride performance. For example, while Glu shows the strongest binding energy (−11.05 eV) and achieves impressive cycling stability (2000 h at 1 mA cm^−2^), some carboxylate‐containing materials (L‐CN and SS) with intermediate binding energies (e.g., −0.496 eV) exhibit comparable or even better cycling performance (under similar conditions of 5200 h) under similar conditions.^[^ ^153^, ^155^, ^163^ ^]^ This non‐linear relationship suggests that optimal zinc protection requires a balanced binding strength rather than simply aiming for the strongest possible interaction.

The comparison among different additive concentrations shown in the figure (ranging from 10 to 500mm) reveals interesting insights into concentration‐performance relationships. For instance, L‐CN and SS at ≈10 mm demonstrate excellent cycling stability (>5000h), while Gly at a much higher concentration (500 mm) shows relatively shorter cycling time.^[^ ^153^, ^155^, ^163^ ^]^ This suggests that higher additive concentrations do not necessarily translate to better performance, challenging the conventional more is better approach. Looking at the current density data at 1 mA cm^−2^, we observe varying performance levels across different functional groups. Under the same cycling conditions, the addition of only 5 mm maltose enabled the battery to hit a cycle life of 5200 h. In contrast, the addition of 500 mm glycine resulted in a cycle life of 3200 h only.^[^ ^155^, ^170^ ^]^ This performance variation likely stems from the different mechanisms by which these functional groups regulate zinc ion transport and deposition processes at the electrode interface. The molecular structures and their corresponding performance metrics suggest that the effectiveness of additives depends more on the optimal balance of concentration and functional group arrangement rather than simply the type or amount of functional groups present.

When comparing single‐group versus multi‐group materials (Table S1, Supporting Information), we observe that the performance enhancement is not merely additive.^[^ ^16^, ^65^, ^66^, ^70^, ^153^, ^154^, ^155^, ^162^, ^163^, ^170^, ^171^, ^172^, ^173^, ^181^, ^182^, ^183^, ^184^, ^185^, ^186^, ^187^ ^]^ From Table S1 (Supporting Information), this can be clearly demonstrated by comparing the performance of different functional group combinations. For instance, taurine (combining ─SO_3_H and ─NH_2_) achieves a remarkable cycling stability of 4000h at 1 mA cm^−2^, while Sodium dodecyl sulfonate containing only sulfonic acid groups has a lifetime of only 2000 h at the same current density.^[^ ^179^, ^181^ ^]^ Similarly, L‐CN (─COOH and ─OH) shows exceptional stability of 5200h, compared to single ─COOH or ─OH group materials which typically show shorter lifespans (e.g., glucose with only ─OH groups shows 2000h). L‐CN, which contains both hydroxyl and carboxyl groups, reduced the interfacial resistance by 80%, in contrast to maltose, which contains only a single hydroxyl group, which reduced it by only 30%.^[^ ^153^, ^170^ ^]^

The spatial distribution of functional groups emerges as a critical yet previously overlooked factor. Our comparative analysis of materials with similar functional group types reveals varying degrees of effectiveness. For example, comparing silk‐derived materials (SP and SS) which share similar functional groups (─COOH and ─NH_2_) but differ in their molecular structure, we observe significant performance variations (3000h vs. 5200h lifespan).^[^ ^154^, ^163^ ^]^ α‐CD, β‐CD, γ‐CD are composed of 6,7,8 glucose heads and tails, respectively, and although the functional groups they contain are identical, the size of the cavity pores of the conical hollow cylinder 3D ring structure formed by spatial factors varies, resulting in differences in the properties of the three substance additives. For example, for hydrogenolysis potential and Tafel slope, α‐CD showed the best results, where the Tafel slope α‐CD (465.9 mV dec^−1^) was much larger than β‐CD (214.1 mV dec^−1^) and γ‐CD (162.4 mV dec^−1^). The adsorption energy is also quite different from that of the Zn(002) crystal surface, −0.87 eV for α‐CD, while β‐CD and γ‐CD are only −0.45 and −0.21 eV.^[^ ^65^ ^]^

Beyond the applications of biomass‐derived materials in aqueous electrolytes (such as ZnSO_4_ or Zn(OTf)_2_) discussed earlier, their roles in hydrogel electrolytes are equally significant, as demonstrated in recent studies. For instance, in the carboxylated double‐network hydrogel electrolyte (Gel/SA‐acetate) composed of gelatin and sodium alginate, the biomass‐derived polymers restructure the solvation sheath of Zn^2+^ ions through a chain‐liquid synergistic effect.^[^ ^188^ ^]^ The abundant carboxyl groups in sodium alginate and hydrophilic chains in gelatin form new hydrogen bonds with water molecules, guiding directional migration of hydrated Zn^2+^ ions and reducing the desolvation energy barrier. This results in a reversible plating/stripping performance for 1580 h in a Zn||Zn symmetric cell and an ultra‐long runtime of 5600 h with a high Coulombic efficiency (99.9%) in an asymmetric cell. In another case, the bio‐inspired biomass hydrogel interface (CCS/SA) constructed from carboxymethyl chitosan and sodium alginate exhibits an ion‐selective responsive sieving mechanism.^[^ ^189^ ^]^ The hydrogel's functional groups (carboxyl, hydroxyl, amino) act as pre‐interception layers to block polyiodide shuttling while facilitating Zn^2+^ transport. The hydrogen bond networks within CCS/SA perturb water activity, suppressing Zn corrosion and HER, enabling a Zn‐I_2_ battery to achieve an unprecedented 60 000 cycles at 5 A g^−1^. These examples highlight that biomass‐derived materials in hydrogel electrolytes not only regulate interfacial ion migration and deposition but also enhance mechanical adaptability and interface stability, offering dual advantages in electrochemical performance and practical applicability.

### 4.2 Comparison of Biomass Materials with Conventional Protectants

Biomass materials have lower costs due to their wide availability (e.g., agricultural and forestry waste) and simple preparation, while traditional protectants are more expensive with complex synthesis processes. In terms of performance, biomass materials show high ionic conductivity, good interfacial compatibility, and stability, whereas some traditional protectants suffer from high interfacial impedance and poor stability. Environmentally, biomass materials are biodegradable and eco‐friendly, unlike non‐degradable traditional protectants that easily cause pollution. A detailed comparision was show in Table S4 of Supporting Information.^[^ ^190^, ^191^, ^192^, ^193^, ^194^ ^]^

## 5 Conclusion and Perspective

This review systematically analyses the recent advances in biomass‐derived interface engineering for zinc metal protection. Through comprehensive examination of structure‐performance relationships, we have identified several key principles governing interface protection mechanisms. The binding energy hierarchy among different functional groups (hydroxyl > sulfonic acid > carboxyl > amino) provides a fundamental basis for material selection, though the relationship between binding strength and performance exhibits notable non‐linearity. More significantly, the synergistic effects of multiple functional groups and their spatial distribution emerge as critical factors that transcend simple additive benefits, offering new strategies for interface engineering.

Despite these advances, several fundamental challenges remain to be addressed. The mechanistic understanding of synergistic effects between functional groups requires deeper investigation, particularly through in situ characterization techniques and molecular dynamics simulations. The development of quantitative models to predict optimal functional group combinations would significantly advance rational material design. Additionally, while many biomass‐derived materials show promising performance, their practical application faces challenges in scalability and cost‐effectiveness. Standardized extraction and modification processes, along with optimization of material usage, are essential for industrial implementation. Looking forward, several critical directions deserve attention for advancing this field.

1. developing advanced characterization techniques, particularly operando methods, will be crucial for understanding interfacial processes at the molecular level. The combination of in situ Raman spectroscopy, atomic force microscopy, and cryogenic electron microscopy could provide unprecedented insights into the dynamic evolution of electrode‐electrolyte interfaces. Integration with computational modelling and machine learning approaches would enable predictive understanding of structure‐performance relationships and accelerate material optimization.
2. systematic investigation of degradation mechanisms is essential for improving long‐term stability. This includes understanding the chemical and structural evolution of interface layers during extended cycling, particularly under extreme conditions. The development of self‐healing interfaces through reversible chemical bonds or dynamic supramolecular interactions represents a promising strategy for achieving sustained protection. Such understanding would guide the design of more robust interface engineering strategies.
3. bio‐inspired design principles could open new frontiers in interface engineering. Natural systems often exhibit sophisticated ion transport and regulation mechanisms that could inform artificial interface design. The integration of multiple protection strategies in hierarchical structures, mimicking biological systems, might provide superior performance. Of particular interest are responsive interfaces that can dynamically adjust their properties based on local electrochemical environments.
4. standardization of evaluation metrics is crucial for meaningful advancement. Beyond conventional cycling tests, comprehensive evaluation systems should incorporate dendrite suppression efficiency, interface stability, and rate capability metrics. Establishing industry‐standard testing protocols and creating systematic structure‐performance databases would accelerate both fundamental research and practical implementation.
5. scaling up biomass‐derived materials while maintaining their performance advantages requires innovative solutions in process engineering. This includes developing efficient extraction methods, ensuring material consistency, and optimizing resource utilization. Success in this area would bridge the gap between laboratory achievements and commercial applications, potentially revolutionizing zinc‐based energy storage technologies.
6. Integrating machine learning (ML) with experimental data to construct predictive models for functional group optimization. ML algorithms can analyze large datasets of binding energies, adsorption behaviors, and cycling performances to identify optimal functional group combinations and spatial arrangements, accelerating the rational design of biomass‐derived materials for zinc protection.
7. natural biomass materials can be optimized via chemical modification to enhance functional group density and spatial arrangement. For example, esterification or amination can introduce more carboxyl or amino groups, while crosslinking can tune pore structures for improved ion transport. Grafting synthetic polymers onto biomass backbones may synergize natural sustainability with tailored electrochemical properties, guiding the design of next‐generation zinc‐protective materials.

The field of biomass‐derived interface engineering for zinc protection has shown remarkable progress, but realizing its full potential requires addressing these challenges through systematic research efforts. Success in these areas could revolutionize zinc‐based energy storage technologies and provide valuable insights for broader electrochemical applications. The integration of multiple protection strategies in hierarchical structures, combined with advanced characterization and computational methods, presents a promising pathway toward next‐generation zinc metal batteries.

## Conflict of Interest

The authors declare no conflict of interest.

## Supporting information



Supporting Information

## Data Availability

The data that support the findings of this study are available from the corresponding author upon reasonable request.
