# Supplementary material for: Structure‐Function Relationships of Biomass‐Derived Materials for Zinc Metal Protection
Source: Adv Sci (Weinh). 2025 Jul 15;12(34):e07768. doi: 10.1002/advs.202507768 (PMC12442658; doi:10.1002/advs.202507768)
Supplement: Supplementary file 1 — Supporting Information [file ADVS-12-e07768-s001.docx]

**Structure-Function Relationships of Biomass-Derived Materials for Zinc Metal Protection**

Qiwei Gao, Zhuo Chen, Ya He, Zhangxiang Hao, Junrun Feng, ^*^

Q. Gao, Z. Chen, Y. He, Z. Hao, J. Feng,

School of Science, School of Chip Industry,

Hubei University of Technology,

Wuhan, Hubei 430068, China

E-mail: fengjunrun@hbut.edu.cn


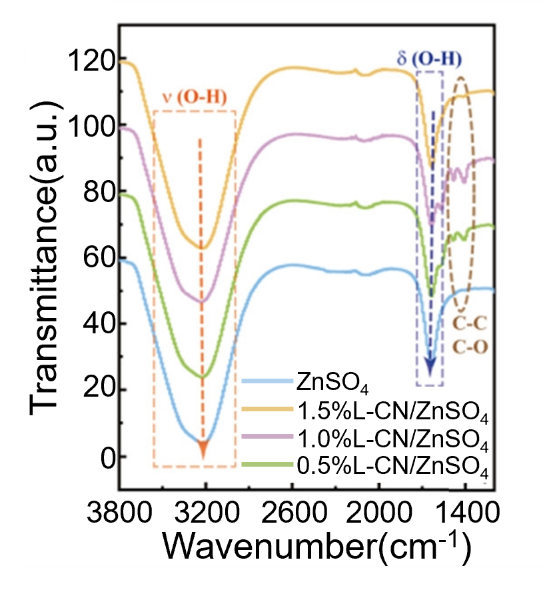


Figure S1. FTIR spectra of different concentrations of L-CN. Reproduced with permission.^[1]^ Copyright 2023, Royal Society of Chemistry.


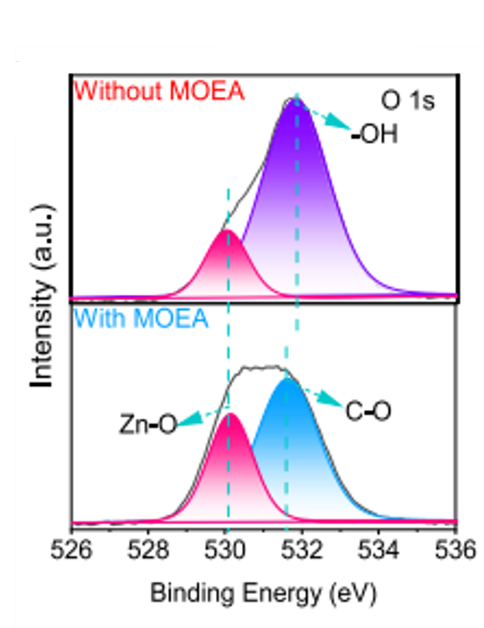


Figure S2. O 1s XPS spectra of Zn deposited in different electrolyte systems. Reproduced with permission.^[2]^ Copyright 2025, American Chemical Society.


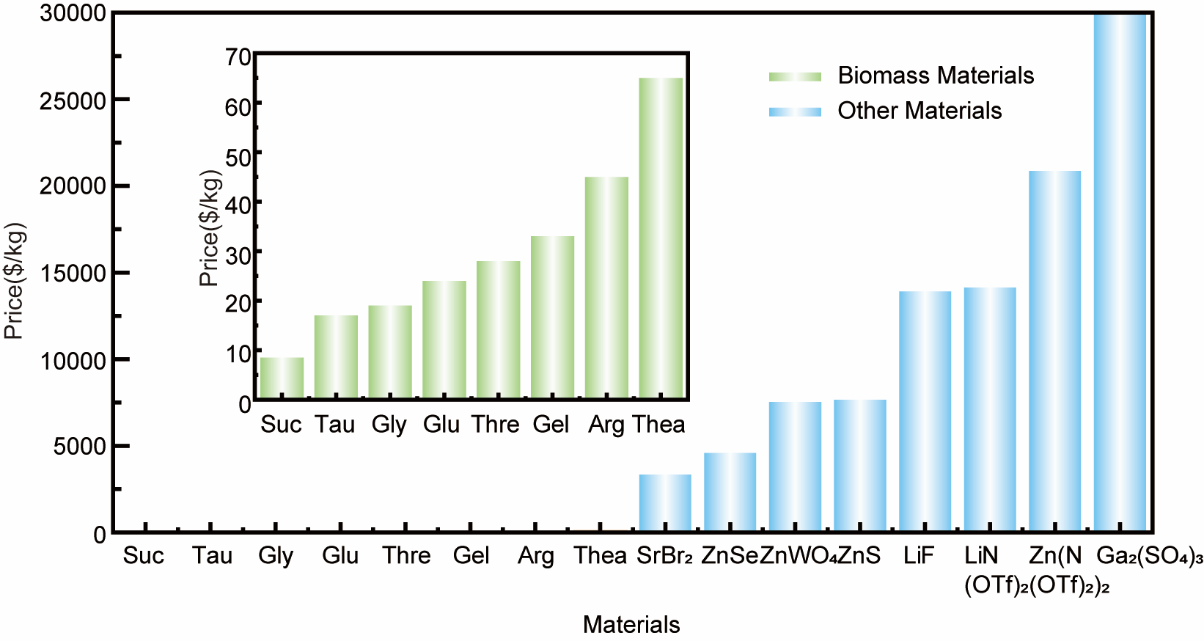


Figure S3. Histogram of prices of biomass and inorganic materials.

Table S1. Summary of the symmetric cell performances with biomass-modified electrolyte.

| Additive | Structure | Functional  Groups | Electrolyte component | Absorption  Energy  (Zn(002))/eV | Binding Energy  (Zn^2+^)/eV | Potential/  V(vs.  Ag/AgCl） | Cycling condition  /(mA cm^-2^-mAh cm^-2^) | Lifespan/h | Ref. |
| --- | --- | --- | --- | --- | --- | --- | --- | --- | --- |
| Glycine | 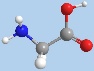 | -COOH;  -NH_2_ | 2M ZnSO₄ +0.5 M Gly | -0.658 | -0.274 | -1.146 | 1-1 | 3200 | ^[3]^ |
|  |  |  |  |  |  |  | 10-1 | 460 |  |
| Arginine | 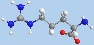 | -COOH;  -NH_2_ | 1M ZnSO₄ +0.1M Arg | -3.85 | - | -1.625 | 1-1 | 2950 | ^[4]^ |
|  |  |  |  |  |  |  | 5-4 | 2200 |  |
|  |  |  |  |  |  |  | 10-4 | 900 |  |
| Threonine | 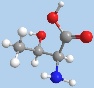 | -COOH;  -NH_2_;-OH | 3M ZnSO₄ +10mM TH | .-1.1(O of COOH);  -0.91(OH of COOH);  -1.01(OH);  -0.86（NH_2_） | - | -0.08V(vs. Zn/Zn^2+^） | 1-1 | 580 | ^[5]^ |
|  |  |  |  |  |  |  | 5-1 | 700 |  |
| Silk Peptide | 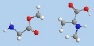 | -COOH;  -NH_2_ | 2M ZnSO₄ +5mg mL^-1^SP | -2.09 | -1.35 (COOH);  -0.86(NH_2_) | -1.11 | 1-1 | 3000 | ^[6]^ |
| Silk Sericin | 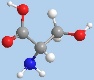 | -COOH;  -NH_2_;-OH | 1M ZnSO₄ + 1% SS | -7.143 | -0.496  (COOH);-0.437(NH_2_) | -180.6mV(vs. Zn/Zn^2+^） | 1-1 | 5200 | ^[7]^ |
| L-carnitine | 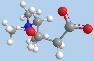 | -COOH;  -OH | 2M ZnSO₄ +1.5%L-CN | -1.54 | -1.56 | - | 1-1 | 5200 | ^[1]^ |
|  |  |  |  |  |  |  | 40-1 | 10000  (cycle number) |  |
|  |  |  |  |  |  |  | 8.85-8.85 | 975 |  |
| Glucose | 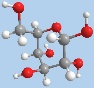 | -OH | 1M ZnSO₄ + 10mM Glu | -1.07 | -11.05 | - | 1-1 | 2000 | ^[8]^ |
|  |  |  |  |  |  |  | 5-5 | 270 |  |
| Trehalose | 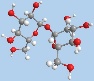 | -OH | 2M ZnSO₄ +100mM Tre | - | -4.16 | -1.05V(vs.SCE) | 2-1 | 1500 | ^[9]^ |
|  |  |  |  |  |  |  | 5-1 | 1600 |  |
| α-cyclodextrin | 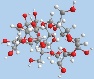 | -OH | 3M ZnSO₄ + 10mM α-CD | -0.87 | - | 1.6V(vs. SCE) | 1-1 | 600 | ^[10]^ |
| Maltose | 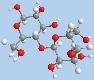 | -OH | 3M ZnSO₄ + 5mM Malt | -4.368 | -6.233 | -1.88 | 2-1 | 5300 | ^[11]^ |
| Xylitol | 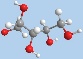 | -OH | 2M ZnSO₄ + 10mM Xylitol | -0.863 | -1.19 | -1.078 | 1-1 | 1100 | ^[12]^ |
|  |  |  |  |  |  |  | 5-1 | 1000 |  |
| Sucrose | 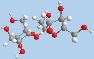 | -OH | 1M ZnSO₄ + 10mM Suc | -1.26 | -7.1 | - | 1-1 | 2000 | ^[13]^ |
|  |  |  |  |  |  |  | 5-5 | 300 |  |
| Taurine | 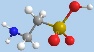 | -SO_3_H;  -NH_2_ | 2M ZnSO₄ + 50mM TA | -0.348 | - | -1.15 | 20-20 | 609 | ^[14]^ |
|  |  |  |  |  |  |  | 40-40 | 209 |  |
| Taurine | 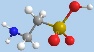 | -SO_3_H;  -NH_2_ | 2M ZnSO₄ + 20mM TA | -2.02 | -2.26 | -1.3 | 1-1 | 4000 | ^[15]^ |
|  |  |  |  |  |  |  | 10-5 | 1000 |  |
| L-cysteine | 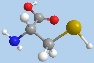 | -COOH;  -NH_2_ | 1M ZnSO₄ + 0.83mM Cys | -2.29(Zn(0001)) | - | -0.99 | 2-2 | 1600 | ^[16]^ |
|  |  |  |  |  |  |  | 10-10 | 200 |  |
| L-asparagine | 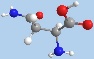 | -COOH;  -NH_2_ | 2M ZnSO₄ + 0.1M Asp | -0.525 | -0.25 | -1.91(vs. Zn/Zn^2+^） | 1-1 | 4260 | ^[17]^ |
| Monosodium Glutamate | 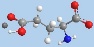 | -COOH;  -NH_2_ | 1M ZnSO₄ + 0.1M MSG | - | - | -0.8 | 1-1 | 4000 | ^[18]^ |
|  |  |  |  |  |  |  | 10-10 | 520 |  |
| Polyamino Acid | 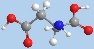 | -COOH | 2M ZnSO₄ + 1% PAA | -2.22 | -4.34 | -1.3 | 1-1 | 2200 | ^[19]^ |
|  |  |  |  |  |  |  | 5-3 | 300 |  |
| Histidine | 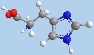 | -COOH;  -NH_2_ | 1M ZnSO₄ + 0.02M HD | -1.16 | - | -1.58 | 2-2 | 3000 | ^[20]^ |
|  |  |  |  |  |  |  | 5-2 | 2300 |  |
|  |  |  |  |  |  |  | 10-4 | 670 |  |
| Theanine | 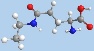 | -COOH;  -NH_2_ | 2M ZnSO₄ + 20mM TN | -0.961 | -0.93 | -1.288 | 5-1 | 2000 | ^[21]^ |

Table S2. Price comparison of biomass materials with inorganic materials.

| Biomass-Derived Materials | Sucrose | Taurine | Glycine | Glucose Anhydrose | L-Threonine | Gelatin | L-Arginine | L-Theanine |
| --- | --- | --- | --- | --- | --- | --- | --- | --- |
| Price($/kg) | 8.5 | 17 | 19 | 24 | 28 | 33 | 45 | 65 |
| Other Materials | SrBr_2_ | ZnSe | ZnWO_4_ | ZnS | LiF | LiN(SO_2_CF_3_)_2_ | Zn(N(SO_2_CF_3_)_2_)_2_ | Ga_2_(SO_4_)_3_ |
| Price($/kg) | 3339 | 4,591 | 7526 | 7,651 | 13,911 | 14134 | 20,866 | 29,908 |

Table S3. Ionic conductivity and interfacial resistance of biomass materials containing hydroxyl groups

| Additive | Trehalose | α-cyclodextrins | Maltose | Xylitol | Glucose |
| --- | --- | --- | --- | --- | --- |
| Electrolyte component | 2M ZnSO_4_+100mM Tre | 3M ZnSO_4_+10mM α-CD | 2M ZnSO_4_+5mM malt | 2M ZnSO_4_+100mM xylitol | 1M ZnSO_4_+10mM Glu |
| group densities(mM L^-1^) | 80 | 180 | 40 | 500 | 50 |
| ionic conductivity(mS cm^-1^) | 106.6 | 58.4 | 14.2 | 75 | 129 |
| interfacial resistance(Ω) | 500 | 300 | 300 | 54 | 200 |

Table S4. Comparison of biomass materials with conventional protectants

| **Dimension** | biomass | **conventional protectant** |
| --- | --- | --- |
| **costs** | Significantly lower, raw materials from renewable resources (e.g. plant, crustacean waste), simple production process. | Highly dependent on fossil fuels or complex synthesis processes. For example, PVDF needs to be prepared by fluorination, which is energy-intensive and expensive as a raw material. |
| **performances** | Cycling Stability: Xanthan Gum-based protective film keeps zinc electrodes stable after 200 days of cycling; Chitosan gel electrolyte achieves 99.7% energy efficiency after 1000 cycles.  Dendrite Inhibition: Xanthan gum combined with ionic conductive polymers form a smooth protective layer that effectively mitigates the growth of zinc dendrites.  Ionic conductivity: Cellulose acetate (CA)-based solid-state electrolytes have an great ionic conductivity. | Cycle life: MnO_2_ cathode capacity retention rate of 68.2% after 80 cycles; PVDF binder battery cycle stability is poor, capacity decay after 500 cycles.  Dendrite problem: traditional electrolyte is easy to cause zinc dendrite growth, and need to rely on complex additives or structural design |
| **environmental impact** | **Degradability: Chitosan electrolyte is completely decomposed within 5 months , xanthan gum production process has low carbon emission and by-products can be recycled.**  **Low toxicity: Biomass materials are naturally non-toxic and no harmful gases are released during the production process.** | Non-degradable: PVDF is difficult to biodegrade and decomposes at high temperatures to release toxic hydrogen fluoride (HF) gas.  Highly polluting: Mining and synthesis of MnO_2_ involves heavy metal pollution and high energy consumption. |
| **Ref.** | **^[22–24]^** | ^[25,26]^ |

**References:**

[1] H. Yu, D. Chen, X. Ni et al.Energy Environmental Science **2023**, 16, 2684.

[2] L. Xiao, J. Sun, M. Wang, S. Zhang, Y. Xu, ACS Applied Materials & Interfaces 2025, 17, 3356

[3] Y. Liu, Y. An, L. Wu et al. ACS Nano **2023,** 17, 552.

[4] H. Lu, X. Zhang, M. Luo et al. Advanced Functional Materials 2021, 31, 2103514.

[5] Z. Miao, Q. Liu, W. Wei, et al. Nano Energy 2022, 97, 107145.

[6] B. Wang, R. Zheng, W. Yang et al. Advanced Functional Materials **2022**, 32, 2112693.

[7] Y. Xiong, W. Teng, Z. Zhao et al. Energy Storage Materials **2025**, 74, 103959.

[8] P. Sun, L. Ma, W. Zhou, M. Qiu, Z. Wang, D. Chao, W. Mai, Angewandte Chemie - International Edition **2021**, 60, 18247.

[9] H. Liu, Z. Xin, B. Cao et al. Advanced Functional Materials **2024**, 34, 2309840.

[10] K. Zhao, G. Fan, J. Liu, et al. Chemical Research in Chinese Universities **2022**, 144, 11129.

[11] Y. Liu, B. Xie, Q. Hu et al. Energy Storage Materials **2024**, 66, 103202.

[12] H. Wang, W. Ye, B. Yin et al. Angewandte Chemie - International Edition **2023**, 62, e202218872.

[13] C. Wang, J. Hou, Y. Gan et al. Journal of Materials Chemistry A **2023**, 11, 8057.

[14] K. Ouyang, S. Chen, W. Ling et al. Angewandte Chemie - International Edition **2023**, 62, e202311988.

[15] G. Duan, Y. Wang, B. Luo, L. Sun, S. Zheng, J. Huang, Z. Ye, Energy Storage Materials **2023**, 61, 102882.

[16] C. Huang, X. Zhao, Y. Hao et al. Small **2022**, 18, 2203674.

[17] X. Wang, K. Feng, B. Sang et al. Advanced Energy Materials **2023**, 13, 2301670.

[18] Y. Liu, J. Wang, J. Sun et al. Journal of Materials Chemistry A **2022**, 10, 25029.

[19] J. Liu, W. Song, Y. Wang et al. Journal of Materials Chemistry A **2022**, 10, 20779.

[20] Z. Zhao, P. Li, Z. Zhang, H. Zhang, G. Li, Chemical Engineering Journal **2023**, 454, 140435.

[21] N. Hu, W. Lv, W. Chen, et al. Advanced Functional Materials **2024**, 34, 2311773.

[22] B. Bhuvaneswari, M. Sivabharathy, L. G. Prasad, S. Selvasekarapandian, Ionics (Kiel) **2022**, 28, 3865.

[23] M. Wu, Y. Zhang, L. Xu et al. Matter **2022**, 5, 3402.

[24] J. Jang, J. Chun, C. Jo, Energy Storage Materials **2023**, 62. 102948.

[25] N. Jaikrajang, W. Kao-Ian, T. Muramatsu et al. ACS Applied Energy Materials **2021**, 4, 7138.

[26] Y. Zhu, Y. Cui, H. N. Alshareef, Nano Letters **2021**, 21, 1446.
